# Long-Term Follow-up Observation of the Safety, Immunogenicity, and Effectiveness of Gardasil™ in Adult Women

**DOI:** 10.1371/journal.pone.0083431

**Published:** 2013-12-31

**Authors:** Joaquin Luna, Manuel Plata, Mauricio Gonzalez, Alfonso Correa, Ivete Maldonado, Claudia Nossa, David Radley, Scott Vuocolo, Richard M. Haupt, Alfred Saah

**Affiliations:** 1 Instituto Nacional de Cancerologia, Bogotá, Colombia; 2 Fundacion Cardioinfantil, Bogotá, Colombia; 3 Clinica del Country, Bogotá, Colombia; 4 Fundacion Santa Fe de Bogotá, Bogotá, Colombia; 5 Cafam, Bogotá, Colombia; 6 Merck Sharp & Dohme Corp., Whitehouse Station, New Jersey, United States of America; University of New South Wales, Australia

## Abstract

**Background:**

Previous analyses from a randomized trial in women aged 24–45 have shown the quadrivalent HPV vaccine to be efficacious in the prevention of infection, cervical intraepithelial neoplasia (CIN) and external genital lesions (EGL) related to HPV 6/11/16/18 through 4 years. In this report we present long term follow-up data on the efficacy, safety and immunogenicity of the quadrivalent HPV vaccine in adult women.

**Methods:**

**Follow-up** data are from a study being conducted in 5 sites in Colombia designed to evaluate the long-term immunogenicity, effectiveness, and safety of the qHPV vaccine in women who were vaccinated at 24 to 45 years of age (in the original vaccine group during the base study [n = 684]) or 29 to 50 years of age (in the original placebo group during the base study [n = 651]). This analysis summarizes data collected as of the year 6 post-vaccination visit relative to day 1 of the base study (median follow-up of 6.26 years) from both the original base study and the Colombian follow-up.

**Results:**

There were no cases of HPV 6/11/16/18-related CIN or EGL during the extended follow-up phase in the per-protocol population. Immunogenicity persists against vaccine-related HPV types, and no evidence of HPV type replacement has been observed. No new serious adverse experiences have been reported.

**Conclusions:**

Vaccination with qHPV vaccine provides generally safe and effective protection from HPV 6-, 11-, 16-, and 18-related genital warts and cervical dysplasia through 6 years following administration to 24–45 year-old women.

**Trial Registration:**

Clinicaltrials.gov NCT00090220

## Introduction

Persistent infection of the uterine cervix by 15 to 20 carcinogenic human papillomavirus (HPV) genotypes leads to the vast majority of cervical cancers [1], [2] and related precursor lesions [3]. While all sexually active women are at risk of HPV infection, the incidence of HPV infection peaks soon after the onset of sexual activity in most populations [4]–[6]. Incidence rates tend to decline thereafter, however some women older than age 25 are likely to remain at significant risk for acquisition of new HPV infections [7], [8].

The quadrivalent HPV (qHPV) (types 6, 11, 16, 18) L1 virus-like particle (VLP) vaccine is highly effective in preventing HPV 6, 11, 16, or 18-related high-grade intraepithelial neoplasia and condyloma in men and women aged 16 to 26 naïve to the respective vaccine HPV types at enrollment [9], [10] In the pivotal FUTURE II trial (1095) 12,167 women between the ages of 15 and 26 received three doses of either HPV-6/11/16/18 vaccine or placebo, administered at day 1, month 2, and month 6. Subjects were followed for an average of 3 years after receiving the first dose of vaccine or placebo. Vaccine efficacy for the prevention of HPV 16/18 disease was 98% (95% CI: 86–100) in the per-protocol susceptible population. In addition, the efficacy of the qHPV vaccine has previously been demonstrated in women 24 to 45 years of age participating in an international double blind clinical trial (FUTURE III) [11]. End of study data (mean follow-up time of 3.8 years) from FUTURE III demonstrated qHPV vaccine efficacy of 88.7% (95% CI: 78.1, 94.8) against the combined incidence of persistent infection, cervical intraepithelial neoplasia (CIN) or external genital lesions (EGL) related to vaccine HPV types in the per-protocol population on women aged 24–45 [12]. Through the end of study follow-up, 91.5%, 92.0%, 97.4% and 47.9% of vaccinated women were still considered seropositive to HPV 6, 11, 16 and 18, respectively.

In this report we elaborate on previous FUTURE III results by presenting the findings of a long-term follow-up of that study evaluating the effectiveness, immunogenicity and safety of the qHPV vaccine in women originally enrolled into the trial from Colombian study sites. Analyses are cumulative incidence since vaccination, and therefore include base study data from all subjects as well as follow-up data from subjects in Colombia only.

## Methods

### Base study design

Between June 18, 2004 and April 30, 2005, 3,819 women between the ages of 24 and 45 years were enrolled at 38 international study sites into a randomized, placebo-controlled, double-blind safety, immunogenicity, and efficacy study (NCT00090220). Subjects were enrolled from community health centers, academic health centers, and primary health care providers in Colombia, France, Germany, Philippines, Spain, Thailand, and the United States. Descriptions of treatments, endpoints, hypotheses and case definitions have been published [11], [12]. The institutional review board (IRB) at each participating center approved the protocol and written informed consent was obtained from all subjects. Studies were conducted in conformance with applicable country or local requirements regarding ethical committee review, informed consent and other statutes or regulations regarding the protection of the rights and welfare of human subjects participating in biomedical research. The names of each participating IRB can be seen in the supplementary file ‘IRB’.

The protocol for this trial and supporting CONSORT checklist are available as supporting information; see Checklist S1 and Protocol S1.

### Follow-up study

V501-Protocol 019-21 is a study being conducted in 5 sites in Colombia and is designed to evaluate the long-term immunogenicity, effectiveness, and safety of V501, the quadrivalent Human Papillomavirus (Types 6, 11, 16, 18) recombinant vaccine (qHPV vaccine) in women who were vaccinated at 24 to 45 years of age (for those enrolled in the original vaccine group during the protocol 019 base study) or 29 to 50 years of age (if they were in the original placebo group during the protocol 019 base study). This interim analysis report summarizes data collected as of the year 6 post-vaccination visit (relative to day 1 of the base study). Future analyses are planned at year 8 and year 10 (end-of-study analysis). The analyses performed are cumulative incidence since vaccination. Base study data from all subjects contributes for the first 4 years. However, only subjects from Colombia contribute follow-up data after 4 years.

This long-term study does not have a placebo group. Subjects who were vaccinated with qHPV vaccine in the base study at 24 to 45 years of age are referred to as the “early vaccination group” (EVG) in this report. Subjects who were vaccinated with placebo in the base study were later vaccinated with a three dose regimen of qHPV vaccine during the first extension of the base study at 29 to 50 years of age (V501 protocol 019-10) and are referred to as the “catch-up vaccination group” (CVG). Because there is no placebo group for the long term follow-up phase, vaccine efficacy cannot be measured. In lieu of efficacy measurements, effectiveness of vaccination with qHPV vaccine is assessed by calculating the incidence of disease endpoints in each of the EVG and CVG and comparing these rates with those observed in groups vaccinated in previous efficacy studies within Merck's qHPV vaccine program. No study vaccinations were provided within the context of this long-term follow-up study.

### Safety Objective

To observationally describe the general safety of a 3-dose regimen of qHPV vaccine [Human Papillomavirus (Types 6, 11, 16, 18) Recombinant Vaccine] in women 24 to 50 years of age during a period of 5 to 10 years following vaccine dose one by describing vaccine- or study procedure-related serious adverse experiences (SAEs), SAEs resulting in death, prespecified medical conditions, and pregnancy follow-up outcomes.

The long-term safety profile will be characterized in terms of incidence of SAEs that a study investigator considers to be possibly, probably, or definitely related to prior administration of the qHPV vaccine or to a study procedure; incidence of death; prespecified medical conditions; and incidence of pregnancy, including pregnancy outcomes and fetal or infant condition.

### Immunogenicity objective

To evaluate the kinetics and age-dependence of anti-HPV 6, 11, 16, and 18 responses following a 3-dose regimen of qHPV vaccine at approximately 6, 8, and 10 years after vaccination dose one among subjects from the qHPV vaccine group of the base study and approximately 1, 3, and 5 years after vaccination dose one among subjects from the placebo group of the base study. The long-term immunogenicity profile will be characterized in terms of geometric means of vaccine-HPV types 6, 11, 16, and 18 titers and seropositivity to the same four vaccine-HPV types. Two different assays will be used; the competitive Luminex immunoassay (cLIA) and the total IgG assay.

### Effectiveness objective

To observationally describe the incidence of HPV 6/11/16/18 related genital warts or cervical dysplasia, and of HPV 16/18-related CIN2+ (CIN grades 2 or 3, cervical AIS, and cervical cancer) up to 6 years following administration of qHPV vaccine in 24 to 50 year-old women. The analyses are cumulative incidence since vaccination. Therefore, the base study data from all subjects contributes for the first 4 years. Only the Colombian subjects have follow-up after 4 years.

HPV type replacement will also be observationally described. The incidence of non-HPV 6/11/16/18 related genital warts or cervical dysplasia, and of non-HPV 16/18-related CIN2+ (CIN grades 2 or 3, adenocarcinoma in-situ, and cervical cancer) will be assessed in Colombian women. The HPV types that will be studied are those studied in the base study, specifically HPV 31/33/35/39/45/51/52/56/58/59 and all high-grade lesions due to any non-vaccine type.

### Populations studied

All subjects who received at least one dose of the qHPV vaccine and have follow-up data, starting from when they have signed consent to enter the long-term study will be included in the summaries of safety.

The primary effectiveness analysis approach for the EVG will be per-protocol-efficacy (PPE), as defined during the base study. To be eligible for this population, subjects must (i) have received 3 doses of GARDASIL™ within one year, and (ii) have no protocol violations. Subjects will be considered cases related to a given HPV type provided the subject was negative to the respective HPV type by serology and PCR prior to vaccination (i.e., during the base study) and PCR negative through month 7. For purposes of endpoint definition, only the Pathology Panel diagnosis will be considered. Supportive analyses will be done for the group naïve to the relevant HPV type (HNRT) and full analysis set (FAS) populations. The HNRT population consists of all subjects who received at least one vaccination and were seronegative and PCR negative to the relevant type prior to vaccination. The FAS population includes all vaccinated subjects, regardless of HPV status.

For the CVG, no serology or swab samples were taken immediately prior to vaccination with qHPV vaccine in the extension. However, any positivity recorded during the base study period (day 1 to month 48) was counted. By comparison, the EVG subjects had a single time-point of assessment for baseline HPV positivity.

### Procedures

No new randomization was performed during this long-term follow-up study. The allocation numbers assigned to study subjects from Colombia during the base study were carried over to this study. At visits corresponding to years 6, 8 and 10 after the beginning of the base study, subjects underwent medical and gynecologic histories, pelvic exams, ThinPrep™ Pap tests and a wart/lesion inspection. In addition, serum samples were taken for anti-HPV serology testing. No new vaccinations were provided in this long-term follow-up study.

Cytology specimens were evaluated using the Bethesda System—2001. For all cytology diagnoses of Atypical Squamous Cells of Undetermined Significance (ASC-US), a central laboratory automatically performed reflex HPV testing on residual ThinPrep™ material, using the Digene Hybrid Capture II™, High Risk/Low Risk Probes. If at least 1 probe was positive or if no result was obtained, the subject was referred for colposcopy. If cervical biopsies and/or endocervical curettage (ECC) specimens were obtained, specimens were sent to the central laboratory for analysis. Endpoint adjudication was performed by an independent pathology panel.

### Competitive Luminex Immunoassay

The competitive Luminex immunoassay (cLIA) [13] simultaneously evaluates the presence of conformational, neutralizing antibodies to the four HPV types present in the quadrivalent vaccine; HPV 6, 11, 16 and 18.(11,27) On each VLP there are neutralizing epitopes that may or may not be HPV type-specific, as well as epitopes that are non-neutralizing.(1,3–8,14,29) The cLIA is a multiplex assay that involves the displacement of a phycoerythrin (PE)-labeled HPV type-specific, VLP-conformation dependent, neutralizing monoclonal antibody (mAb): H6.M48, K11.B2, H16.V5, and H18.J4 for the HPV 6, 11, 16, and 18 assays, respectively.(11,27) Luminex microspheres are coated with HPV type 6, 11, 16 and 18 L1 VLPs and incubated with the PE-labeled mAbs. Displacement of the PE-labeled mAb is an indirect measure of human serum antibody binding to the monitored neutralizing epitopes relative to the reference standard. Human serum was diluted 1∶4 and tested. Titers are reported in arbitrary cLIA milliMerck Units per milliliter (cLIA mMU/mL) determined by the MFI correlation to the reference standard interpolated through a four parameter curve-fitting algorithm. As a unique reference standard curve is generated for each HPV type, and because each HPV type employs a type-specific mAb with a unique binding affinity, the recorded cLIA mMU/mL titers are not equivalent and cannot be directly compared between HPV types.

### Total IgG Luminex Immunoassay

A total IgG Luminex immunoassay (LIA) was developed utilizing yeast-derived L1 VLPs of HPV types 6, 11, 16 and 18 coupled to a set of distinct fluorescent Luminex microspheres [14]. Samples were tested in duplicate in this validated research assay and titers determined relative to a 12-point standard curve. The median fluorescent intensity (MFI) of the bound phycoerythrin (PE) tagged mouse anti-human IgG monoclonal antibody clone HP6043 (Biotrend, Destin, FL) was captured as the raw VLP-specific bound total IgG direct binding data. This mAb binds equally to all four human IgG isotypes, IgG1-IgG4.(17) The correlation of MFI units to an arbitrary IgG milliMerck Unit per milliliter (IgG mMU/mL) of VLP-specific IgG was made by serially diluting the reference serum and interpolating the MFI data through a 4-parameter curve fitting algorithm.

### Statistical analysis

In the absence of a control group in the long-term follow-up study, it is not possible to perform analyses of efficacy. Instead, effectiveness will be measured in terms of incidence rates of disease, with analyses performed periodically until the end of the follow-up. Incidence rates based on accrued person-years of follow-up will be estimated together with a corresponding 95% confidence interval, calculated using the exact confidence limits of the binomial distribution, as an approximation to the Poisson distribution. Analyses will combine events and follow-up time from the base study and, for those participating, from the extension study. Exact binomial confidence intervals were calculated for the proportions of subjects seropositive

## Results

**Figure 1:**
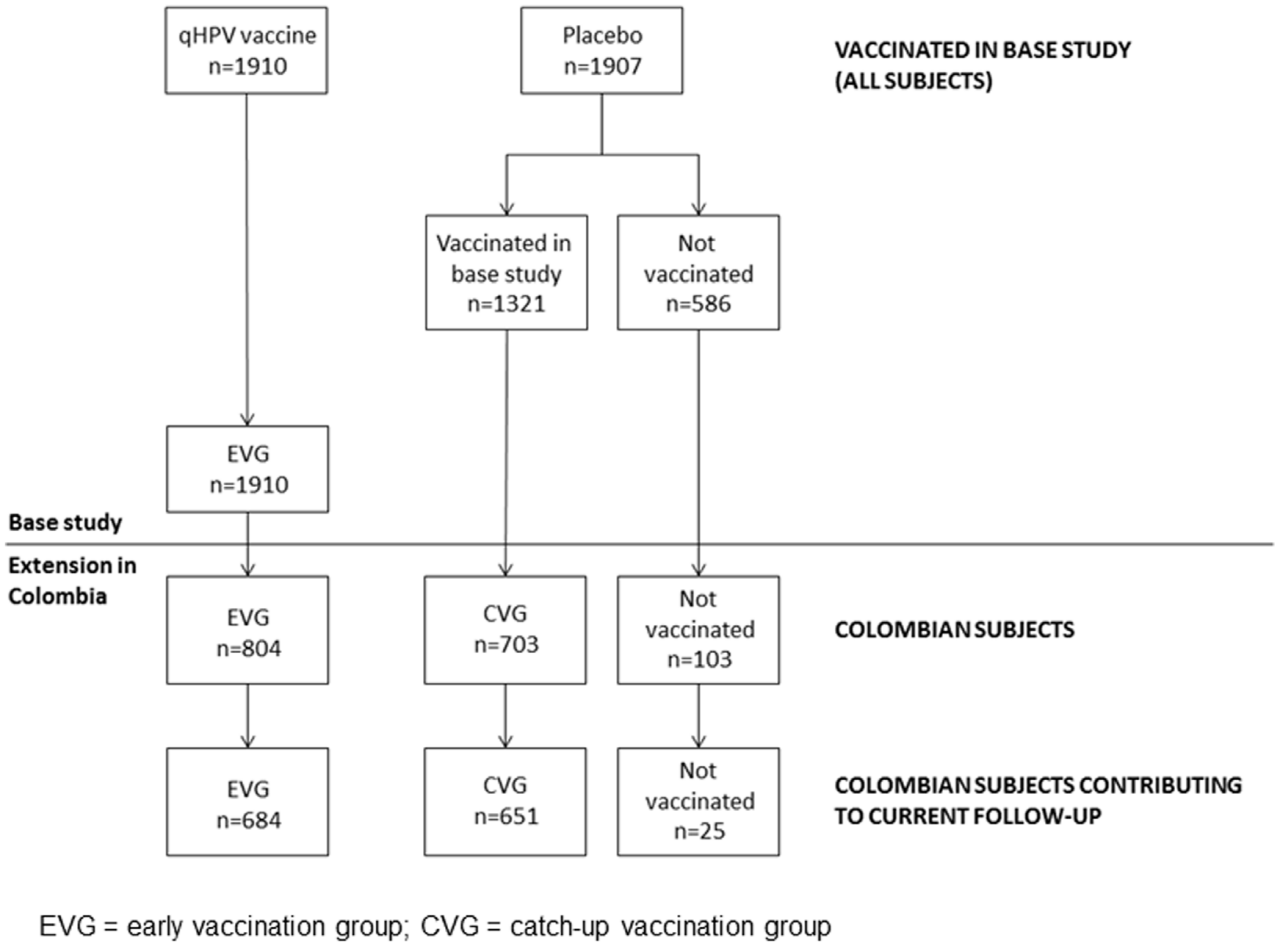
Subject accounting in base study and extension. 804 EVG subjects and 703 CVG subjects took part in the Colombian extension study. Data were available for 684 subjects in the EVG and 651 CVG subjects.

Figure 1 displays the subject disposition in the base study and its extension. A total of 3,817 study subjects were randomized in a 1∶1 ratio and vaccinated with either qHPV vaccine (N = 1,910) or placebo (N = 1,907), in the context of the base study. This extension study is being conducted only in Colombia. Enrollment into the base study in Colombia was 1,610 in total (804 randomized to and vaccinated with qHPV vaccine, 806 randomized to and vaccinated with placebo). A total of 1,360 Colombian subjects participated in this extension (84% of the subjects enrolled in base study in that country).

This report is comprised of follow-up data from study start through the cutoff date of 02-Nov-2011. The median follow-up time from day 1 of the base study in the EVG was 6.26 years. For the CVG who received qHPV vaccine after the base study, the median follow-up time post-dose 1 of the vaccine was 1.18 years. Table 1 displays selected subject characteristics at the time of first vaccination for subjects enrolled in Colombia. The difference in age between the groups reflects vaccination of the CVG at the end of the base study.

**Table 1:** Baseline characteristics of subjects in Colombia at the time of vaccination dose 1 in the base study.

	Early Vaccination	Catch-up Vaccination	
	Group	Group	Total
Characteristic at Day 1 (qHPV)	(N = 804)	(N = 703)	(N = 1,507)
**Gender - % (m)**			
Female	100% (804)	100% (703)	100% (1,507)
**Age (years)**			
Mean	34.7	39.8	37.1
Standard Deviation	6.3	6.0	6.7
Median	35	40	38
Range	24 to 45	29 to 50	24 to 50
24 to 34 years old	47.5% (382)	24.6% (173)	36.8% (555)
35 to 45 years old	52.5% (422)	53.6% (377)	53.0% (799)
>45 years old	0.0% (0/0)	21.8% (153)	10.2% (153)
**Race - % (m)**			
Black	0.5% (4)	0.4% (3)	0.5% (7)
Hispanic American	99.5% (800)	99.6% (700)	99.5% (1,500)
**Serostatus** † **- % (m/n)**			
Positive to HPV 6/11/16/18	29.9% (240/804)	47.4% (333/703)	38.0% (573/1,507)
Positive to HPV 6	14.1% (113/804)	27.6% (194/703)	20.4% (307/1,507)
Positive to HPV 11	5.0% (40/804)	12.7% (89/703)	8.6% (129/1,507)
Positive to HPV 16	14.9% (120/804)	28.9% (203/703)	21.4% (323/1,507)
Positive to HPV 18	5.1% (41/804)	5.0% (35/703)	5.0% (76/1,507)
**PCR status** † **- % (m/n)**			
Positive to HPV 6/11/16/18	9.3% (74/797)	24.3% (171/703)	16.3% (245/1,500)
Positive to HPV 6	1.6% (13/796)	8.4% (59/703)	4.8% (72/1,499)
Positive to HPV 11	0.4% (3/796)	0.9% (6/703)	0.6% (9/1,499)
Positive to HPV 16	5.2% (41/796)	13.7% (96/703)	9.1% (137/1,499)
Positive to HPV 18	2.4% (19/797)	5.7% (40/703)	3.9% (59/1,500)

Day 1 (qHPV) is the day of injection of dose 1 of the qHPV vaccine.

Unless otherwise indicated the percents shown were calculated as 100*(m/N).

N = Number of subjects in the indicated vaccination group who received at least 1 dose of the qHPV vaccine.

n = Number of subjects with non-missing Day 1 (qHPV) status corresponding to the indicated HPV type.

m = Number of subjects belonging to the indicated category.

qHPV = Quadrivalent Human Papillomavirus [Types 6, 11, 16, 18] Recombinant Vaccine.

For Catch-Up Vaccination Group, positivity at any time between Day 1 and Month 48 is used.

The rates of positivity prior to vaccination in the CVG are generally higher than those observed in the EVG at the start of the base study, partly as a result of increased ascertainment of infection and partly because of persistent risk of infection in this older age group of women.

Table 2 displays the cumulative incidence of HPV 6/11/16/18-related genital warts or cervical dysplasia, from the start of the base study through all visits completed before the cutoff date, in the EVG cohort. There was a single case of this endpoint, an HPV 16-related CIN2, observed in the PPE population during the base study. To date, no additional cases of this endpoint have occurred in follow-up visits of the EVG PPE population. Four (4) cases of HPV 6/11/16/18-related genital warts or cervical dysplasia have occurred in the EVG HNRT population (Table 2); all had been reported during the base study. Compared to the per-protocol population, this population additionally includes protocol violators, including those receiving less than 3 vaccinations, and those who became HPV-positive at or prior to month 7 of the base study. In follow-up visits to date, no additional cases of this endpoint have occurred in the EVG HNRT population. A total of 36 cases of HPV 6/11/16/18-related genital warts or cervical dysplasia have been observed in the EVG FAS population (Table 2); all had been reported during the base study. Compared to the HNRT population, this population additionally includes subjects positive to the relevant HPV type at day 1 of the base study.

**Table 2:** Effectiveness of qHPV vaccination in women 24–45 years of age against HPV 6/11/16/18-related CIN or condyloma (cumulative incidence in the EVG, day 1 to year 6).

	Early Vaccination Group (N = 1,910)				
	n	Cases	PYR	Rate	95% CI
**Per-protocol population (PPE)**					
HPV 6/11/16/18-Related CIN or Condyloma	1,617	1	6,705.6	0.0	(0.0, 0.1)
By HPV Type					
HPV 6-Related CIN or Condyloma	1,330	0	5,515.4	0.0	(0.0, 0.1)
HPV 11-Related CIN or Condyloma	1,330	0	5,515.4	0.0	(0.0, 0.1)
HPV 16-Related CIN or Condyloma	1,351	1	5,611.5	0.0	(0.0, 0.1)
HPV 18-Related CIN or Condyloma	1,524	0	6,314.1	0.0	(0.0, 0.1)
By Endpoint Type (HPV 6/11/16/18-Related)					
CIN (any grade)	1,599	1	6,349.8	0.0	(0.0, 0.1)
CIN 1	1,599	0	6,352.4	0.0	(0.0, 0.1)
CIN 2 or worse	1,599	1	6,349.8	0.0	(0.0, 0.1)
CIN 2	1,599	1	6,349.8	0.0	(0.0, 0.1)
CIN 3	1,599	0	6,352.4	0.0	(0.0, 0.1)
AIS	1,599	0	6,352.4	0.0	(0.0, 0.1)
Cervical Cancer	1,599	0	6,352.4	0.0	(0.0, 0.1)
Condyloma	1,617	0	6,696.8	0.0	(0.0, 0.1)
**Naïve to the relevant type population (HNRT)**					
HPV 6/11/16/18-Related CIN or Condyloma	1,863	4	8,511.0	0.0	(0.0, 0.1)
By HPV Type					
HPV 6-Related CIN or Condyloma	1,535	1	7,041.1	0.0	(0.0, 0.1)
HPV 11-Related CIN or Condyloma	1,535	0	7,041.6	0.0	(0.0, 0.1)
HPV 16-Related CIN or Condyloma	1,572	3	7,187.2	0.0	(0.0, 0.1)
HPV 18-Related CIN or Condyloma	1,760	0	8,052.8	0.0	(0.0, 0.0)
By Endpoint Type (HPV 6/11/16/18-Related)					
CIN (any grade)	1,862	3	8,108.1	0.0	(0.0, 0.1)
CIN 1	1,862	1	8,110.7	0.0	(0.0, 0.1)
CIN 2 or worse	1,862	3	8,109.8	0.0	(0.0, 0.1)
CIN 2	1,862	3	8,109.8	0.0	(0.0, 0.1)
CIN 3	1,862	1	8,112.3	0.0	(0.0, 0.1)
AIS	1,862	0	8,112.4	0.0	(0.0, 0.0)
Cervical Cancer	1,862	0	8,112.4	0.0	(0.0, 0.0)
Condyloma	1,863	1	8,509.0	0.0	(0.0, 0.1)
**Full analysis set population (FAS)**					
HPV 6/11/16/18-Related CIN or Condyloma	1,910	36	8,601.8	0.4	(0.3, 0.6)
By HPV Type					
HPV 6-Related CIN or Condyloma	1,910	10	8,702.7	0.1	(0.1, 0.2)
HPV 11-Related CIN or Condyloma	1,910	1	8,728.9	0.0	(0.0, 0.1)
HPV 16-Related CIN or Condyloma	1,910	25	8,639.0	0.3	(0.2, 0.4)
HPV 18-Related CIN or Condyloma	1,910	3	8,724.9	0.0	(0.0, 0.1)
By Endpoint Type (HPV 6/11/16/18-Related)					
CIN (any grade)	1,909	29	8,303.6	0.3	(0.2, 0.5)
CIN 1	1,909	17	8,310.6	0.2	(0.1, 0.3)
CIN 2 or worse	1,909	21	8,311.9	0.3	(0.2, 0.4)
CIN 2	1,909	11	8,313.7	0.1	(0.1, 0.2)
CIN 3	1,909	16	8,317.1	0.2	(0.1, 0.3)
AIS	1,909	0	8,319.2	0.0	(0.0, 0.0)
Cervical Cancer	1,909	0	8,319.2	0.0	(0.0, 0.0)
Condyloma	1,910	7	8,698.1	0.1	(0.0, 0.2)

N = Number of subjects in the indicated group who received at least 1 dose of the qHPV vaccine.

n = Number of subjects in the indicated analysis population.

PYR = person years at risk; Rate = rate per 100 person years at risk; AIS = Adenocarcinoma in situ; CI = Confidence interval; CIN = Cervical intraepithelial neoplasia; HPV = Human papillomavirus; qHPV = Quadrivalent Human Papillomavirus (Types 6, 11, 16, 18) Recombinant Vaccine.

The cumulative incidence of non-vaccine HPV type (HPV 31/33/35/39/45/51/52/56/58/59)-related genital warts or cervical dysplasia, from the start of the base study through all visits completed before the cutoff date, in the EVG FAS population can be seen in Table 3. Of the total 93 cases of this endpoint, a single case has been reported subsequent to the base study (an HPV 31-related CIN1 lesion).

**Table 3:** Effectiveness of qHPV vaccination in women 24 to 45 years of age against HPV 31/33/35/39/45/51/52/56/58/59-related CIN or condyloma (cumulative incidence, day 1 to year 6 in the EVG FAS population).

	Early Vaccination Group (N = 1,910)				
Endpoint	n	Cases	PYR	Rate	95% CI
HPV 31/33/35/39/45/51/52/56/58/59-Related CIN or Condyloma	1,910	93	8,403.7	1.1	(0.9, 1.4)
By HPV Type					
HPV 31-Related CIN or Condyloma	1,910	16	8,678.1	0.2	(0.1, 0.3)
HPV 33-Related CIN or Condyloma	1,910	7	8,711.7	0.1	(0.0, 0.2)
HPV 35-Related CIN or Condyloma	1,910	4	8,717.4	0.0	(0.0, 0.1)
HPV 39-Related CIN or Condyloma	1,910	17	8,668.3	0.2	(0.1, 0.3)
HPV 45-Related CIN or Condyloma	1,910	5	8,720.4	0.1	(0.0, 0.1)
HPV 51-Related CIN or Condyloma	1,910	17	8,686.5	0.2	(0.1, 0.3)
HPV 52-Related CIN or Condyloma	1,910	13	8,698.5	0.1	(0.1, 0.3)
HPV 56-Related CIN or Condyloma	1,910	23	8,654.5	0.3	(0.2, 0.4)
HPV 58-Related CIN or Condyloma	1,910	17	8,664.4	0.2	(0.1, 0.3)
HPV 59-Related CIN or Condyloma	1,910	6	8,712.4	0.1	(0.0, 0.1)
HPV 31/33/35/39/45/51/52/56/58/59-Related CIN or Condyloma	1,910	93	8,403.7	1.1	(0.9, 1.4)
By Endpoint Type					
CIN (any grade)	1,909	93	8,191.1	1.1	(0.9, 1.4)
CIN 1	1,909	72	8,210.3	0.9	(0.7, 1.1)
CIN 2 or worse	1,909	40	8,296.4	0.5	(0.3, 0.7)
CIN 2	1,909	28	8,302.2	0.3	(0.2, 0.5)
CIN 3	1,909	23	8,311.0	0.3	(0.2, 0.4)
AIS	1,909	0	8,319.2	0.0	(0.0, 0.0)
Cervical Cancer	1,909	0	8,319.2	0.0	(0.0, 0.0)
Condyloma	1,910	0	8,721.0	0.0	(0.0, 0.0)

N = Number of subjects in the indicated group who received at least 1 dose of the qHPV vaccine.

n = Number of subjects in the indicated analysis population.

PYR = person years at risk; Rate = rate per 100 person years at risk; AIS = Adenocarcinoma in situ; CI = Confidence interval; CIN = Cervical intraepithelial neoplasia; HPV = Human papillomavirus; qHPV = Quadrivalent Human Papillomavirus (Types 6, 11, 16, 18) Recombinant Vaccine.

Table 4 displays the incidence of the vaccine type-related composite effectiveness endpoints in 2-year intervals covering the base study and follow-up period. This table provides context for the incidence of these endpoints observed in the follow-up period to date for the EVG and CVG (no data are available for the CVG from years 4–6) in both the PPE and FAS populations. For HPV 6/11/16/18-related CIN or condyloma (Table 4A), the incidence rate within the PPE CVG during the base study was 0.4/100 person-years for day 1 to year two, and 0.4/100 person-years for year two to year four. By comparison, the two-year incidence rate in year four to year six in the PPE EVG was 0.0/100 person-years, with an upper 95% confidence limit of 0.3. No cases have been reported during extension study visits.

**Table 4:** Comparison of 2-year incidence rates of vaccine and non-vaccine HPV type-related endpoints.

A. Per-protocol efficacy (PPE) population (vaccine HPV types only)											
		Early Vaccination Group					Catch-up Vaccination Group				
		(N = 1,910)					(N = 1,907)				
Endpoint	Period	n	Cases	P-Y	Rate	95% CI	n	Cases	P-Y	Rate	95% CI
HPV 6/11/16/18-Related CIN or Condyloma											
	Day 1 - Year 2	1602	1	2276	0.0	(0.0, 0.2)	1599	8	2265	0.4	(0.2, 0.7)
	Year 2 - Year 4	1559	0	3024	0.0	(0.0, 0.1)	1550	11	2990	0.4	(0.2, 0.7)
	Year 4 - Year 6	927	0	1226	0.0	(0.0, 0.3)	-	-	-	-	-
HPV 16/18-Related CIN2 or Worse											
	Day 1 - Year 2	1570	1	2191	0.0	(0.0, 0.3)	1558	2	2174	0.1	(0.0, 0.3)
	Year 2 - Year 4	1483	0	2828	0.0	(0.0, 0.1)	1475	4	2806	0.1	(0.0, 0.4)
	Year 4 - Year 6	842	0	1109	0.0	(0.0, 0.3)	-	-	-	-	-
HPV 6/11-Related Condyloma											
	Day 1 - Year 2	1316	0	1872	0.0	(0.0, 0.2)	1316	3	1868	0.2	(0.0, 0.5)
	Year 2 - Year 4	1285	0	2491	0.0	(0.0, 0.1)	1285	4	2484	0.2	(0.0, 0.4)
	Year 4 - Year 6	751	0	1002	0.0	(0.0, 0.4)	-	-	-	-	-

B. Full analysis set (FAS) population.											
		Early Vaccination Group					Catch-up Vaccination Group				
		(N = 1,910)					(N = 1,907)				
Endpoint	Period	n	Cases	P-Y	Rate	95% CI	n	Cases	P-Y	Rate	95% CI
HPV 6/11/16/18-Related CIN or Condyloma											
	Day 1 to Year 2	1886	29	3627	0.8	(0.5, 1.1)	1884	41	3614	1.1	(0.8, 1.5)
	Year 2 to Year 4	1757	6	3402	0.2	(0.1, 0.4)	1744	19	3352	0.6	(0.3, 0.9)
	Year 4 to Year 6	1040	1	1371	0.1	(0.0, 0.4)	-	-	-	-	-
HPV 16/18-Related CIN2 or Worse											
	Day 1 to Year 2	1862	17	3577	0.5	(0.3, 0.8)	1862	19	3573	0.5	(0.3, 0.8)
	Year 2 to Year 4	1712	4	3259	0.1	(0.0, 0.3)	1706	8	3236	0.2	(0.1, 0.5)
	Year 4 to Year 6	978	0	1284	0.0	(0.0, 0.3)	-	-	-	-	-
HPV 6/11-Related Condyloma											
	Day 1 to Year 2	1884	5	3662	0.1	(0.0, 0.3)	1883	8	3657	0.2	(0.1, 0.4)
	Year 2 to Year 4	1781	2	3449	0.1	(0.0, 0.2)	1776	4	3426	0.1	(0.0, 0.3)
	Year 4 to Year 6	1041	0	1390	0.0	(0.0, 0.3)	-	-	-	-	-
HPV 31/33/35/39/45/51/52/56/58/59-Related CIN or Condyloma											
	Day 1 to Year 2	1886	63	3593	1.8	(1.3, 2.2)	1884	61	3595	1.7	(1.3, 2.2)
	Year 2 to Year 4	1727	28	3312	0.8	(0.6, 1.2)	1725	24	3308	0.7	(0.5, 1.1)
	Year 4 to Year 6	1002	1	1304	0.1	(0.0, 0.4)	-	-	-	-	-
HPV 31/33/35/39/45/51/52/56/58/59-Related CIN2 or Worse											
	Day 1 to Year 2	1862	32	3570	0.9	(0.6, 1.3)	1862	18	3573	0.5	(0.3, 0.8)
	Year 2 to Year 4	1707	6	3254	0.2	(0.1, 0.4)	1707	6	3237	0.2	(0.1, 0.4)
	Year 4 to Year 6	976	2	1280	0.2	(0.0, 0.6)	-	-	-	-	-

N = Number of subjects in the indicated group who received at least 1 dose of the qHPV vaccine.

P-Y = person years; Rate = rate per 100 person years at risk; CI = Confidence interval; CIN = Cervical intraepithelial neoplasia.

Similar data were seen for vaccine-related CIN or condyloma in the FAS population (Table 4B). For non-vaccine (HPV 31/33/35/39/45/51/52/56/58/59)-related disease, the incidence rate within the FAS CVG during the base study was 1.7/100 person-years for day 1 to year 2, and 0.7/100 person-years for year 2 to year 4. By comparison, the incidence rate in year 4 to year 6 in the EVG is 0.1, with an upper 95% confidence limit of 0.4.

The proportion of subjects who are seropositive to HPV 6, 11, 16 and 18 at month 72 (year 6) from first vaccination, as measured by cLIA and total IgG assays can be seen in Table 5. Month 7 cLIA seropositivity for HPV types 6, 11, 16 and 18 was 97.6%, 97.0%, 97.9% and 96.8%, respectively (data not shown). While some declines are seen in seropositivity after the vaccination course, month 72 cLIA seropositivity was comparable to that observed at month 48. As noted in previous studies, the proportion of subjects seropositive to HPV 18 by the cLIA declines over time because of the nature of the assay. At month 72, overall seropositivity is 45%, whereas for HPV 6, 11 and 16, seropositivity is maintained at approximately 90% or higher. Of note, no cases of HPV 18-related disease have been observed in the EVG PPE population, during the base study or during follow-up to date.

**Table 5:** Summary of month 72 HPV seropositivity rates by age (per-protocol immunogenicity population participating in follow-up).

	Early Vaccination Group (N = 1,910)					
	24 to 34 Year-olds		35 to 45 Year-olds		All Subjects	
	(N = 953)		(N = 957)		(N = 1,910)	
Assay (cLIA)		SPR		SPR		SPR
	n	(95% CI)	N	(95% CI)	n	(95% CI)
**Anti-HPV 6**						
cLIA	198	89.4% (84.2%, 93.3%)	270	88.9% (84.5%, 92.4%)	468	89.1% (85.9%, 91.8%)
Total IgG	198	87.9% (82.5%, 92.1%)	270	87.8% (83.3%, 91.4%)	468	87.8% (84.5%, 90.6%)
**Anti-HPV 11**						
cLIA	196	94.4% (90.2%, 97.2%)	270	90.4% (86.2%, 93.6%)	466	92.1% (89.2%, 94.3%)
Total IgG	198	87.4% (81.9%, 91.7%)	270	82.2% (77.1%, 86.6%)	468	84.4% (80.8%, 87.6%)
**Anti-HPV 16**						
cLIA	197	97.5% (94.2%, 99.2%)	276	97.1% (94.4%, 98.7%)	473	97.3% (95.3%, 98.5%)
Total IgG	197	100% (98.1%, 100%)	276	99.6% (98.0%, 100%)	473	99.8% (98.8%, 100%)
**Anti-HPV 18**						
cLIA	237	48.5% (42.0%, 55.1%)	293	42.7% (36.9%, 48.5%)	530	45.3% (41.0%, 49.6%)
Total IgG	237	84.4% (79.1%, 88.8%)	293	79.2% (74.1%, 83.7%)	530	81.5% (77.9%, 84.7%)

N = Number of subjects in the indicated group who received at least 1 dose of the qHPV vaccine.

n = Number of subjects with non-missing titer in the indicated analysis population.

CI = Confidence interval; cLIA = Competitive Luminex immunoassay; total IgG = total IgG assay.

Month 72 total IgG seropositivity for HPV 6, 11, 16 and 18 was 87.8%, 84.4%, 98.8% and 81.5%, respectively. It is noticeable that the overall seropositivity for HPV 18 exceeds 80% (Table 5), which is much higher than cLIA seropositivity and due to the particular features of each assay. Subjects 24 to 34 years of age appear to have a slightly higher response to all HPV types in this assay, compared to subjects 35 to 45 years of age, although the 95% CI for age-specific GMTs overlap.

No serious adverse experiences have been reported in the context of the long-term extension study period. Table S1 displays outcomes for pregnancies reported in the long-term extension study period, after the base study. Of the four known outcomes to date, all have resulted in normal live births. Approximately 13% of all subjects had at least one new medical condition reported in the long-term extension study period. The most commonly reported new medical conditions were bacterial vaginitis, hypothyroidism, and uterine leiomyoma. The number of subjects with cancer or conditions of potential autoimmune etiology was low. The two vaccination groups were generally well balanced with regard to the proportions of subjects reporting specific categories of medical conditions.

## Discussion

Previous reports have demonstrated the efficacy, safety and immunogenicity of the qHPV vaccine in women aged 24 to 45 [11], [12]. While these earlier reports had a maximum mean follow-up time of just under 4 years; the current analysis extends this follow-up to a mean of 6.26 years.

There were no cases of HPV 6/11/16/18-related CIN or condyloma, HPV 16/18-related CIN 2 or worse or HPV 6/11-related condyloma during the follow-up visits in years 4 to 6 in all of the analysis populations. This finding suggests that qHPV vaccine effectiveness continues to be high in this population of women through 6 years following vaccination. The observed person-time of follow-up after the base study was not large enough to demonstrate statistically significant protection against HPV 16/18-related CIN 2 or worse and HPV 6/11-related condyloma. However, the upper 95% CI of the incidence of HPV 6/11/16/18-related CIN or condyloma was below the 4-year incidence of the same endpoint in the placebo group of the base study.

There was no evidence of HPV type replacement in those women who were vaccinated with qHPV vaccine. Accordingly, the incidence of non-vaccine HPV types did not increase proportionally over time.

The immunogenicity data from the cLIA showed persisting high titers over 72 months and showed no significant reduction in GMT from month 48 to month 72. Similarities in GMTs between the two age strata across all HPV types and time points could be seen when comparing the data over 72 months. While some differences between the two age strata can be seen, these differences are small and the 95% CIs overlap. Additionally, when the entire PPI population and those women who entered the long-term follow-up segment of the study are compared, the GMTs are similar for all HPV types and time points.

No significant reduction in seropositivity from month 48 to month 72 was seen. Moreover, seropositivity at month 72 is similar to month 24 for each of the HPV types, including HPV 18. This observation indicates that effectiveness against HPV 18 related disease persisted when the cLIA was positive in approximately 50% of women for the last two years of the base study and for the first two years of the long-term follow-up study.

As described [15], [16], the nature of the cLIA in measuring antibodies (including HPV 18) is a function of the monoclonal antibody used in the competitive assay. When the total IgG assay is used, the proportion of women who are positive at month 72 is substantially higher (81.5% overall). Despite these immunogenicity findings, effectiveness continued to be observed by the sustained absence of disease due to HPV 18, as well as due to the other HPV vaccine types.

A drawback of the current study is its localization to subjects enrolled only from Colombia. While the data are interesting and observational in nature, the generalizability of the findings would be greater if enrolling a larger and/or more geographically diverse cohort.

Administration of the qHPV vaccine in the base study was generally well tolerated. Moreover, no serious adverse experiences have been reported in the context of the long-term extension study period. The proportion of subjects who reported serious adverse experiences was comparable between the qHPV vaccine group and the placebo group. Of the four known pregnancy outcomes to date, all have resulted in normal live births.

It is clear that HPV vaccination is likely to be beneficial to sexually active adult women as they are at risk of acquiring new HPV infection and related sequelae [8]. However, public health recommendations for mass vaccination must take into consideration the cost-effectiveness of vaccination programs. Current vaccine and implementation cost modelling studies have shown that vaccination becomes less cost-effective with the increasing age of the target vaccination group, likely due to prior HPV exposure in this case. Since the overall cost–benefit becomes progressively less favorable with increasing age, most health authorities have not widely recommended routine vaccination of older women. Nevertheless, as documented in this trial and others, sexually active women over the age of 26 also have the potential to benefit from vaccination and should be allowed the opportunity to choose to be vaccinated on an individual basis.

In summary, we have demonstrated that vaccination with qHPV vaccine provides durable protection from HPV 6-, 11-, 16-, and 18-related genital warts and cervical dysplasia through 6 years following administration to 24–45 year-old women. In addition, the qHPV vaccine shows no tendency to select for disease due to HPV types that are not present in the vaccine through 6 years following vaccination in women 24 to 45 years of age. Lastly, administration of qHPV vaccine to women 24 to 45 years of age is generally safe and well tolerated through year 6 post-vaccination.

## Supporting Information

Table S1 Pregnancy outcomes. There were 4 live births in the EVG population and none in the CVG population. No fetal losses have occurred.
(DOC)

Checklist S1 CONSORT Checklist.
(DOC)

Protocol S1 Trial Protocol.
(DOC)
