# Supplementary material for: Long-Term Follow-up Observation of the Safety, Immunogenicity, and Effectiveness of Gardasil™ in Adult Women
Source: PLoS One. 2013 Dec 31;8(12):e83431. doi: 10.1371/journal.pone.0083431 (PMC3877052; doi:10.1371/journal.pone.0083431)
Supplement: Table S1 — Pregnancy outcomes. There were 4 live births in the EVG population and none in the CVG population. No fetal losses have occurred. (DOC) [file pone.0083431.s001.doc]

**Table S1**. Pregnancy outcomes

|  | Early Vaccination Group | | Catch-up Vaccination Group | |
| --- | --- | --- | --- | --- |
|  | (N=1910) | | (N=1321) | |
|  | n | (%) | n | (%) |
| Number of Subjects | 1910 |  | 1321 |  |
| Subjects with pregnancies | 5 | (0.3) | 5 | (0.4) |
| Subjects without pregnancies | 1905 | (99.7) | 1316 | (99.6) |
|  |  |  |  |  |
| Number of pregnancies ^†^ | 5 |  | 5 |  |
| Number of pregnancies with unknown outcome | 1 |  | 5 |  |
| Number of pregnancies with known outcome | 4 |  | 0 |  |
|  |  |  |  |  |
| **Live Births^‡^** | 4 | (100.0) | 0 | (0.0) |
|  |  |  |  |  |
| *Method of Delivery* |  |  |  |  |
| C-Section ^§^ | 2 | (50.0) | 0 | (0.0) |
| Other | 2 | (50.0) | 0 | (0.0) |
| Vaginal | 2 | (50.0) | 0 | (0.0) |
|  |  |  |  |  |
| *Infant Outcome* |  |  |  |  |
| Normal | 4 | (100.0) | 0 | (0.0) |
| Abnormal | 0 | (0.0) | 0 | (0.0) |
| Congenital Anomaly | 0 | (0.0) | 0 | (0.0) |
| Other Abnormality | 0 | (0.0) | 0 | (0.0) |
| Unknown | 0 | (0.0) | 0 | (0.0) |
|  |  |  |  |  |
| **Fetal Loss^‡^** | 0 | (0.0) | 0 | (0.0) |
|  |  |  |  |  |
| *Type of Loss* |  |  |  |  |
| Ectopic Pregnancy | 0 | (0.0) | 0 | (0.0) |
| Spontaneous Abortion | 0 | (0.0) | 0 | (0.0) |
| Late Fetal Death | 0 | (0.0) | 0 | (0.0) |
| Elective Abortion | 0 | (0.0) | 0 | (0.0) |
|  |  |  |  |  |
| *Fetal Outcome* |  |  |  |  |
| Normal | 0 | (0.0) | 0 | (0.0) |
| Abnormal | 0 | (0.0) | 0 | (0.0) |
| Congenital Anomaly | 0 | (0.0) | 0 | (0.0) |
| Other Abnormality | 0 | (0.0) | 0 | (0.0) |
| Unknown | 0 | (0.0) | 0 | (0.0) |
| ^†^ A subject may have more than one pregnancy during the study. Each pregnancy is counted once. A pregnancy with multiple fetuses is counted as a single pregnancy, but outcome for each fetus/infant is counted individually.  ^‡^ Percentages of 'Live Births', 'Fetal Loss' and 'Ectopic Pregnancy' are calculated based on the number of fetuses/infants with known outcome. Percentages under 'Method of Delivery' and 'Infant Outcome' are calculated based on 'Live Births'. Percentages under 'Type of Loss' and 'Fetal Outcome' are calculated based on 'Fetal Loss'.  ^§^ A subject may have more than one reason for C-section for a single pregnancy.  N = Number of vaccinated subjects in each vaccination group.  Infant and Fetus outcomes are counted according to the following criteria: Normal if Congenital Anomaly is No AND Describe Other Abnormality is blank; Abnormal if Congenital Anomaly is Yes OR Describe Other Abnormality is not blank; Unknown if Congenital Anomaly is Unknown AND Describe Other Abnormality is blank. | | | | |
