# Supplementary material for: Long-Term Follow-up Observation of the Safety, Immunogenicity, and Effectiveness of Gardasil™ in Adult Women
Source: PLoS One. 2013 Dec 31;8(12):e83431. doi: 10.1371/journal.pone.0083431 (PMC3877052; doi:10.1371/journal.pone.0083431)
Supplement: Protocol S1 — Trial Protocol. (DOC) [file pone.0083431.s003.doc]

**THIS PROTOCOL AND ALL OF THE INFORMATION RELATING TO IT ARE CONFIDENTIAL AND PROPRIETARY PROPERTY OF MERCK SHARP & DOHME CORP., A SUBSIDIARY OF MERCK & CO., INC., WHITEHOUSE STATION, NJ, U.S.A.**

**THIS PROTOCOL REPLACES THE ORIGINAL PROTOCOL AND ANY SUBSEQUENT AMENDMENTS AND SHOULD BE SIGNED BY ALL INVESTIGATORS SIGNING THE ORIGINAL PROTOCOL.**

SPONSOR:

Merck Sharp & Dohme Corp., a subsidiary of Merck & Co., Inc. (hereafter referred to as the **SPONSOR** or Merck)

One Merck Drive

P.O. Box 100

Whitehouse Station, NJ, 08889-0100, U.S.A.

Protocol-specific Sponsor Contact information can be found in the Administrative Binder.

TITLE:

Long Term Follow-up Study of Safety, Immunogenicity, and Effectiveness of GARDASIL™ (Human Papillomavirus [Types 6, 11, 16, 18] Recombinant Vaccine) in Mid-Adult Women—The FUTURE III (Females United to Unilaterally Reduce Endo/Ecto Cervical Cancer) Study

INVESTIGATOR:

**PRIMARY:**

**CLINICAL PHASE:** III

**US IND NUMBER:** 9030

SITE:

INSTITUTIONAL REVIEW BOARD/ETHICS REVIEW COMMITTEE:

Summary of Changes

PRIMARY REASON FOR THIS AMENDMENT:

To clarify guidance criteria used to determine vaccine-relatedness of an adverse event. This change was previously reported in a protocol clarification letter dated 22-Sep-2010.

Sections impacted:

- Table 3-2 in section 3.4.6 was updated to move "possibly related" from the, "No, there is not a reasonable possibility of vaccine relationship", category and placed into the, "Yes, there is a reasonable possibility of vaccine relationship", category.

OTHER CHANGES INCLUDED IN THE AMENDMENT:

No other changes were made.

Table of Contents

Summary of Changes [2](#__RefHeading___Toc274236453)

PRIMARY REASON FOR THIS AMENDMENT: [2](#__RefHeading___Toc274236454)

OTHER CHANGES INCLUDED IN THE AMENDMENT: [2](#__RefHeading___Toc274236455)

1. SUMMARY [7](#__RefHeading___Toc274236456)

1.1 Title [7](#__RefHeading___Toc274236457)

1.2 Indication for Long term Follow-Up [7](#__RefHeading___Toc274236458)

1.3 Summary of Rationale [7](#__RefHeading___Toc274236459)

1.4 Summary of Study Design [7](#__RefHeading___Toc274236460)

1.5 Sample [8](#__RefHeading___Toc274236461)

1.6 Dosage/Dosage Form, Route, and Dose Regimen [8](#__RefHeading___Toc274236462)

1.7 Study Flow Chart [9](#__RefHeading___Toc274236463)

2. CORE PROTOCOL [10](#__RefHeading___Toc274236464)

2.1 Objectives and Hypotheses [10](#__RefHeading___Toc274236465)

2.1.1 Primary Safety [10](#__RefHeading___Toc274236466)

2.1.2 Immunogenicity [10](#__RefHeading___Toc274236467)

2.1.3 Effectiveness [10](#__RefHeading___Toc274236468)

2.2 Subject/Patient Inclusion Criteria [11](#__RefHeading___Toc274236469)

2.3 Subject/Patient Exclusion Criteria [11](#__RefHeading___Toc274236470)

2.4 Study Design and Duration [11](#__RefHeading___Toc274236471)

2.4.1 Summary of Study Design [11](#__RefHeading___Toc274236472)

2.4.2 Treatment Plan [12](#__RefHeading___Toc274236473)

2.5 List of Effectiveness/Immunogenicity Measurements [12](#__RefHeading___Toc274236474)

2.5.1 Immunogenicity Measurements [12](#__RefHeading___Toc274236475)

2.5.2 Effectiveness Measurements [12](#__RefHeading___Toc274236476)

2.5.3 HPV Type-Specific Replacement Measurements [13](#__RefHeading___Toc274236477)

2.6 List of Safety Measurements [14](#__RefHeading___Toc274236478)

2.7 Statistical Analysis Plan Summary [14](#__RefHeading___Toc274236479)

3. PROTOCOL DETAILS [16](#__RefHeading___Toc274236480)

3.1 Rationale [16](#__RefHeading___Toc274236481)

3.1.1 Long Term Follow-Up [16](#__RefHeading___Toc274236482)

3.2 Study Procedures [16](#__RefHeading___Toc274236483)

3.2.1 Concomitant Medication(s)/Treatment(s) [16](#__RefHeading___Toc274236484)

3.2.2 Activity and Other Prerequisites [16](#__RefHeading___Toc274236485)

3.2.3 Procedures [17](#__RefHeading___Toc274236486)

3.2.3.1 Informed Consent [17](#__RefHeading___Toc274236487)

3.2.3.2 Randomization/Allocation [18](#__RefHeading___Toc274236488)

3.2.3.3 Treatment/Evaluation/Follow-Up Procedures [18](#__RefHeading___Toc274236489)

3.2.3.3.1 Summary of Scheduled Study Visit Procedures [18](#__RefHeading___Toc274236490)

3.2.3.3.2 Procedures During Pregnancy and Lactation [19](#__RefHeading___Toc274236491)

3.2.3.3.3 External Genital Inspection [20](#__RefHeading___Toc274236492)

3.2.3.3.3.1 Genital Wart/Lesion Treatment and the Event of Recurrence [20](#__RefHeading___Toc274236493)

3.2.3.3.4 Optional Tests for Clinical Symptoms and Clinical Abnormalities [20](#__RefHeading___Toc274236494)

3.2.3.3.4.1 Optional Tests for Clinical Symptoms [20](#__RefHeading___Toc274236495)

3.2.3.3.4.2 Clinical Abnormalities [21](#__RefHeading___Toc274236496)

3.2.3.3.5 Pap Test for Liquid Cytology (ThinPrep™) [21](#__RefHeading___Toc274236497)

3.2.3.3.6 Serum for Antibody Measurements [22](#__RefHeading___Toc274236498)

3.2.3.3.7 Colposcopy/Biopsy/ Definitive Therapy Procedures [22](#__RefHeading___Toc274236499)

3.2.3.3.7.1 Triage for Abnormal Pap Tests [22](#__RefHeading___Toc274236500)

3.2.3.3.7.2 Colposcopy [23](#__RefHeading___Toc274236501)

3.2.3.3.7.3 Procedures for Biopsy and Definitive Therapy [24](#__RefHeading___Toc274236502)

3.2.3.4 Blinding/Unblinding [29](#__RefHeading___Toc274236503)

3.2.3.5 Discontinuation/Withdrawal from Study [30](#__RefHeading___Toc274236504)

3.2.4 STUDY DURATION AND SUBMISSION OF DATA [30](#__RefHeading___Toc274236505)

3.2.4.1 Subject Retention and Relocation [30](#__RefHeading___Toc274236506)

3.3 Efficacy/Pharmacokinetic/Immunogenicity, etc. Measurements [30](#__RefHeading___Toc274236507)

3.3.1 Definition of the Effectiveness and HPV Type Replacement Endpoints [30](#__RefHeading___Toc274236508)

3.3.2 Blinding of HPV Results [30](#__RefHeading___Toc274236509)

3.3.3 Immunogenicity [31](#__RefHeading___Toc274236510)

3.3.4 PCR Assays to Detect HPV in Clinical Specimens [32](#__RefHeading___Toc274236511)

3.3.4.1 Multiplex PCR Assays [32](#__RefHeading___Toc274236512)

3.3.4.2 Quantitative PCR Assays [33](#__RefHeading___Toc274236513)

3.3.5 PCR Assays to Detect HPV in Thinsections of Biopsy Tissue [33](#__RefHeading___Toc274236514)

3.3.6 Preparation and Disposition of Thinsections of Biopsy Tissue [33](#__RefHeading___Toc274236515)

3.3.7 Conduct of the Clinical Trial [34](#__RefHeading___Toc274236516)

3.3.7.1 Responsibility of the Steering Committee [34](#__RefHeading___Toc274236517)

3.3.7.2 Adjudication Procedures [35](#__RefHeading___Toc274236518)

3.4 Safety Measurements [35](#__RefHeading___Toc274236519)

3.4.1 Clinical and Laboratory Measurements for Safety [35](#__RefHeading___Toc274236520)

3.4.2 Recording Adverse Experiences [35](#__RefHeading___Toc274236521)

3.4.3 Definition of an Overdose for This Protocol [36](#__RefHeading___Toc274236523)

3.4.3.1 Reporting of Overdose to SPONSOR [36](#__RefHeading___Toc274236524)

3.4.4 Reporting of Pregnancy to SPONSOR [36](#__RefHeading___Toc274236525)

3.4.5 Immediate Reporting of Adverse Experiences to the SPONSOR [36](#__RefHeading___Toc274236526)

3.4.5.1 Serious Adverse Experiences [36](#__RefHeading___Toc274236527)

3.4.6 Evaluating Adverse Experiences [37](#__RefHeading___Toc274236528)

3.4.7 SPONSOR Responsibility for Reporting Adverse Experiences [40](#__RefHeading___Toc274236529)

3.5 Data Analysis [40](#__RefHeading___Toc274236530)

3.5.1 Responsibility for Analyses/In-House Blinding [40](#__RefHeading___Toc274236531)

3.5.2 Hypotheses/Estimation [41](#__RefHeading___Toc274236532)

3.5.3 Analysis Endpoints [41](#__RefHeading___Toc274236533)

3.5.4 Analysis Populations [41](#__RefHeading___Toc274236534)

3.5.5 Statistical Methods [41](#__RefHeading___Toc274236535)

3.5.5.1 Statistical Methods for Assessment of Effectiveness [42](#__RefHeading___Toc274236536)

3.5.5.2 Statistical Methods for Assessment of HPV type Replacement [44](#__RefHeading___Toc274236537)

3.5.5.3 Statistical Methods for Assessment of Immunogenicity [45](#__RefHeading___Toc274236538)

3.5.6 Multiplicity [45](#__RefHeading___Toc274236539)

3.5.7 Power and Sample Size [45](#__RefHeading___Toc274236540)

3.6 Data Management [48](#__RefHeading___Toc274236541)

3.6.1 Study Documentation and Records Retention [48](#__RefHeading___Toc274236542)

3.6.2 Laboratory Results [48](#__RefHeading___Toc274236543)

3.7 Biological Specimens [48](#__RefHeading___Toc274236544)

3.7.1 Shipment [48](#__RefHeading___Toc274236545)

4. ADMINISTRATIVE AND REGULATORY DETAILS [49](#__RefHeading___Toc274236546)

4.1 Confidentiality [49](#__RefHeading___Toc274236547)

4.1.1 Confidentiality of Data [49](#__RefHeading___Toc274236548)

4.1.2 Confidentiality of Subject/Patient Records [49](#__RefHeading___Toc274236549)

4.1.3 Confidentiality of Investigator Information [49](#__RefHeading___Toc274236550)

4.2 Compliance with Law, Audit, and Debarment [50](#__RefHeading___Toc274236551)

4.3 Compliance with Financial Disclosure Requirements [51](#__RefHeading___Toc274236552)

4.4 Quality Control and Quality Assurance [52](#__RefHeading___Toc274236553)

4.5 Compliance with Information Program on Clinical Trials for Serious or Life Threatening Conditions [52](#__RefHeading___Toc274236554)

4.6 Publications [52](#__RefHeading___Toc274236555)

5. LIST OF REFERENCES [54](#__RefHeading___Toc274236556)

6. APPENDICES [55](#__RefHeading___Toc274236557)

7. ATTACHMENTS [56](#__RefHeading___Toc274236558)

# 1. SUMMARY

## 1.1 Title

Long Term Follow-up Study of Safety, Immunogenicity, and Effectiveness of GARDASIL™ (Human Papillomavirus [Types 6, 11, 16, 18] Recombinant Vaccine) in Mid-Adult Women—The FUTURE III (Females United to Unilaterally Reduce Endo/Ecto Cervical Cancer) Study

## 1.2 Indication for Long term Follow-Up

HPV type-specific effectiveness endpoints will be collected by active surveillance of Pap abnormalities, biopsy and EEC specimens, and definitive therapy specimens using the clinic and investigators in Bogota, Colombia with all specimens tested as in Protocol 019 base study.

## 1.3 Summary of Rationale

Results of the Phase III studies have demonstrated that GARDASIL™ is highly efficacious in preventing HPV 6-, HPV 11-, HPV 16-, and HPV 18-related CIN, AIS, cervical cancer (via CIN 2/3 and AIS), VIN, VaIN, condylomata acuminata, vulvar cancer (via VIN 2/3), and vaginal cancer (via VaIN 2/3) in the group that received GARDASIL™ compared to the placebo group in subjects who were not infected with the relevant HPV type at the start of the study. This long term follow-up study is being conducted to assess the safety, immunogenicity, and effectiveness in the population of women who were vaccinated at 24-45 years of age through 10 years following vaccination Dose 1 (among GARDASIL™ recipients in the base study); and those vaccinated at 29-50 years of age through 5 years following vaccination Dose 1 (among placebo recipients in the base study).

Colombia was selected to perform this study because of the high numbers of study participants recruited from Bogota, and the commitment of both the investigators and the study participants to the study. The investigators also have experience in performing longitudinal studies and in motivating women to maintain adherence to the visit schedule.

## 1.4 Summary of Study Design

This Long Term Follow-up (LTFU) is a safety, immunogenicity, and effectiveness observational study. All subjects will sign an informed consent prior to initiation of any study procedure. Subjects will have been enrolled in the Protocol 019 base study in Colombia. Approximately 1,600 healthy women who meet the eligibility criteria will be invited to participate. The age of the women at the time of vaccination Dose 1 will range from 24 to 45 years of age among GARDASIL™ recipients in the base study and 29 to 50 years of age among placebo recipients in the base study.

HPV-related analyses will include: HPV type-specific PCR-based assays to determine the causal HPV type within a clinical lesion (such as a genital wart or a CIN2+ lesion).

The study will have general guidelines for referral of subjects for colposcopy and biopsy during the follow-up period. All biopsy specimens will also undergo HPV DNA analysis using Thinsection PCR. All biopsies will be analyzed at the central laboratory designated by the SPONSOR. The diagnoses provided by the lab will be used for routine management of subjects. However, the diagnoses provided by the lab will not represent the official diagnoses for study purposes. The slides will subsequently be reviewed by an Independent Pathology Panel; the consensus diagnosis of the Independent Pathology Panel will represent the final diagnosis for study purposes.

## 1.5 Sample

The study will enroll ~1,600 subjects who were enrolled in the Protocol 019 base study in Colombia.

• Cohort 1: ~800 of whom were vaccinated with GARDASIL™ at the beginning of the base study and

• Cohort 2: ~800 of whom were vaccinated with GARDASIL™ during the 019-10 extension (i.e., after the conclusion of the base study).

## 1.6 Dosage/Dosage Form, Route, and Dose Regimen

No vaccination will be provided in the long term follow-up.

## 1.7 Study Flow Chart

| Event/Test | Contact and Obtain Consent | Years following Base Study Day 1 visit (window for each visit is ± 6 months) | | |
| --- | --- | --- | --- | --- |
|  |  | 6 | 8 | 10 |
| Obtain informed consent | x |  |  |  |
| Review inclusion/exclusion criteria |  | x |  |  |
| Provide Participant Identification Card |  | x |  |  |
| Collect medical/gynecologic history ^†^ |  | x | x | x |
| Perform physical exam |  | x | x | x |
| Inspect external genital areas for warts/lesions^‡^ |  | x | x | x |
| Perform pelvic exam |  | x | x | x |
| Perform **optional** tests, as necessary^§^ |  | (x) | (x) | (x) |
| Perform Pap test (ThinPrep™) for cytology || |  | x | x | x |
| Inspect vaginal area for warts/lesions^‡^ |  | x | x | x |
| Perform bimanual exams |  | x | x | x |
| Collect blood (serum) for anti-HPV |  | x | x | x |
| Document clinical follow-up for safety ^¶^ |  | x | x | x |
| **Procedures must be conducted in the order provided within the study flow chart.**  Contact and consent may be obtained any time prior to procedures at Year 6 visit (including the day of year 6 visit). Visits for Year 6, Year 8 and Year 10 will be calculated from Day 1 of original study and visit window for each visit is +/- 6 months of actual time point.  ^†^ History will be updated as needed at these visits and at unscheduled visits.  ^‡^ The external genital wart/lesion exam must be conducted in 2 parts in order to limit contamination between anatomical areas. The external portion of the exam including vulvar, labia, perineum, perianal and other external areas will be performed prior to the pelvic exam. The vaginal portion of the wart/lesion inspection will be performed after the pelvic exam and all specimen collection is completed.  ^§^ HSV Culture may be performed if indicated. Vaginal pH, wet mount, whiff test, and KOH may be performed at the investigator's discretion.  || Cytology for Pap test to be performed by central laboratory selected by SPONSOR.  ^¶^ Serious adverse experiences, as defined in Section 3.4 will be collected for all subjects throughout the extension phase. All reports of pregnancy will be reported to the SPONSOR and followed to outcome. | | | | |

# 2. CORE PROTOCOL

## 2.1 Objectives and Hypotheses

The purpose of this extension is to observe the long term effectiveness of quadrivalent HPV (qHPV) vaccine, GARDASIL™.

### 2.1.1 Primary Safety

Objective: To observationally describe the general safety of a 3-dose regimen of GARDASIL™ [Human Papillomavirus (Types 6, 11, 16, 18) Recombinant Vaccine] in women 24 to 50 years of age during a period of 5 to 10 years following vaccine Dose 1 by describing vaccine- or study procedure-related SAEs, SAEs resulting in death and pre-specified medical conditions, and pregnancy follow-up outcomes.

Hypothesis: No hypothesis relating to safety will be tested.

### 2.1.2 Immunogenicity

Objective: To evaluate the kinetics and age-dependence of anti-HPV 6, 11, 16, and 18 responses following a 3-dose regimen of GARDASIL™ at approximately 6, 8, and 10 years after vaccination Dose 1 among subjects from the qHPV vaccine group of the base study and approximately 1, 3, and 5 years after vaccination Dose 1 among subjects from the placebo group of the base study.

Hypothesis: No hypothesis relating to immunogenicity will be tested.

### 2.1.3 Effectiveness

Objective: To observationally describe the incidence of HPV 6/11/16/18‑related genital warts or cervical dysplasia, and of HPV 16/18-related CIN2+ (Cervical Intraepithelial Neoplasia [CIN] grades 2 or 3, cervical Adenocarcinoma in Situ [AIS], and cervical cancer) 6, 8 and 10 years following administration of GARDASIL™ in 24 to 45 year-old women; and 1, 3, and 5 years following administration of GARDASIL™ in 29 to 50 year-old women.

HPV type replacement will also be observationally described. The incidence of non-HPV 6/11/16/18‑related genital warts or cervical dysplasia, and of non-HPV 16/18-related CIN2+ (Cervical Intraepithelial Neoplasia [CIN] grades 2 or 3, cervical Adenocarcinoma in Situ [AIS], and cervical cancer) in 6, 8, and 10 years following administration of GARDASIL™ in 24 to 45 year-old women; and at 1, 3 and 5 years following administration of GARDASIL™ in 29 to 50 year-old women in Colombia. The HPV types that will be studied are those studied in the base study, specifically HPV 31/33/35/39/45/51/52/56/58/59 and for all high grade lesions due to any non-vaccine type [1]. By way of background, the biological behavior of HPV makes replacement by non-vaccine genotypes a remote possibility according to presentations by both Eduardo Franco and Joakim Dillner at the recent EUROGIN meeting (Monte Carlo, Monaco, (Session DE 1, Vaccination, page 21, Thursday, 18 February 2010, <http://www.eurogin.com/2010/finalprogram.pdf>). Both presentations are in keeping with a summary by Dr. Dillner in [2] (page 202). The biological characteristics of HPV do not lend themselves to type replacement. Namely, HPV uses human polymerase which produces very few mutations to facilitate natural selection, and infection with HPV is characterized by multiple HPV types, which is inconsistent with viral competition – a necessary feature for type replacement.

Hypothesis: No hypothesis relating to effectiveness will be tested.

## 2.2 Subject/Patient Inclusion Criteria

Subject was enrolled in Protocol 019 in Colombia and received at least 1 injection of GARDASIL™ or placebo.

Subject is aware of duration of study and is willing to participate through Year 10 of the study.

## 2.3 Subject/Patient Exclusion Criteria

Subject has any condition which in the opinion of the investigator might interfere with the evaluation of the study objectives.

Subject is unable to give informed consent.

Subject is concurrently enrolled in a clinical study or studies that involve or would interfere with the collection of cervical specimens.

## 2.4 Study Design and Duration

### 2.4.1 Summary of Study Design

Approximately 1,600 healthy women, previously enrolled in Protocol 019 (the base study) and/or Protocol 019-10 in Colombia, will sign an informed consent prior to initiation of any study procedure. The women were 24 to 45 years of age when enrolled in the base study. During the base study at Day 1, subjects were stratified by age into 2 groups: those 24 to 34 years of age and those 35 to 45 years of age. Stratification accomplished balanced allocation within each stratum. In Colombia, approximately 800 subjects were vaccinated with GARDASIL™ during the base study at Day 1 and approximately 800 base study placebo recipients were vaccinated with GARDASIL™ at the end of the base study at approximately Year 5 in accordance with Protocol 019-10.

Safety data following administration of GARDASIL™ were collected on all study subjects within the context of Protocol 019 base study and Protocol 019-10 extension. Investigator-reported serious adverse event data as well as pregnancy outcomes will be collected within the context of this study (Protocol 019-20). The response of the immune system to the vaccine will be measured with a multiplex, competitive Luminex Immunoassay (anti-HPV 6, 11, 16, and 18 cLIA) and other serological assays. The PCR assays used to identify effectiveness endpoints are described in Section 3.3.4. The composition and operation of the blinded Independent Pathology Panel as well as the Panels roles and responsibilities are described in Section 3.3.7.2. The pathology endpoints will be determined by the Independent Pathology Panel, as in the base study.

### 2.4.2 Treatment Plan

No study vaccinations will occur within the context of Protocol 019-20.

## 2.5 List of Effectiveness/Immunogenicity Measurements

### 2.5.1 Immunogenicity Measurements

Serum will be analyzed to assess long term kinetics of immune responses and to explore the relationship between long term immune response and breakthrough disease. Immunogenicity endpoints are:

- Serum geometric mean titers (GMTs)
- Seropositivity rates to HPV 6, 11, 16, and 18

### 2.5.2 Effectiveness Measurements

**External Genital Warts or Cervical Dysplasia related to HPV 6, 11, 16, or 18:**

A subject will be considered to have a case of external genital warts or cervical dysplasia, related to HPV 6, 11, 16, or 18 during the follow-up period if she has the following:

- On an individual biopsy or excised tissue, a consensus by the Independent Pathology Panel diagnosis of condyloma acuminata (genital warts), or on an individual biopsy, ECC, LEEP, or Conization (cold knife/laser) specimen a consensus by the Independent Pathology Panel diagnosis of CIN 1, CIN 2, CIN 3, cervical adenocarcinoma in situ (AIS), or cervical cancer

AND

- Detection of HPV 6, 11, 16, 18 by Thinsection PCR in an adjacent section from the same tissue block.

**Cervical Intraepithelial Neoplasia Grades 2 or 3 (CIN 2+), Adenocarcinoma in Situ (AIS), and/or Cervical Cancer related to HPV 16 or 18:**

A subject will be considered to have a case of CIN 2+, AIS, and/or cervical cancer related to HPV 16 or 18 during the follow-up period if she has the following:

- On an individual biopsy, ECC, LEEP, or Conization (cold knife/laser) specimen, a consensus by the Independent Pathology Panel diagnosis of CIN 2, CIN 3, AIS, or cervical cancer

AND

- Detection of HPV 16 or 18 by Thinsection PCR in an adjacent section from the same tissue block.

### 2.5.3 HPV Type-Specific Replacement Measurements

**External Genital Warts or Cervical Dysplasia related to a specific non-vaccine HPV Type:**

A subject will be considered to have a case of external genital warts or cervical dysplasia related to a specific non-vaccine HPV type during the follow-up period if she has the following:

- On an individual biopsy or excised tissue, a consensus by the Independent Pathology Panel diagnosis of condyloma acuminata (genital warts), or on an individual biopsy, ECC, LEEP, or Conization (cold knife/laser) specimen a consensus by the Independent Pathology Panel diagnosis of CIN 1, CIN 2, CIN 3, cervical adenocarcinoma in situ (AIS), or cervical cancer

AND

- Detection of the relevant (from any of HPV types 31, 33, 35, 39, 45, 51, 52, 56, 58, or 59) HPV type by Thinsection PCR in an adjacent section from the same tissue block.

**CIN 2+, AIS, or Cervical Cancer related to a specific non-HPV 16/18 Type:**

A subject will be considered to have a case of CIN2+, AIS, and/or cervical cancer related to a specific non-vaccine HPV 16/18 type during the follow-up period if she has the following:

- On an individual biopsy, ECC, LEEP, or Conization (cold knife/laser) specimen, a consensus by the Independent Pathology Panel diagnosis of CIN 2, CIN 3, AIS, or cervical cancer

AND

- Detection of the relevant (from any of HPV types 31, 33, 35, 39, 45, 51, 52, 56, 58, or 59) HPV type by Thinsection PCR in an adjacent section from the same tissue block.

## 2.6 List of Safety Measurements

The following safety measurements will be collected:

- Serious Adverse Experiences (SAEs) (as defined in the detailed protocol) judged by the study investigator to be possibly, probably, or definitely related to prior administration of GARDASIL™
- SAEs (as defined in the detailed protocol) judged by the study investigator to be possibly, probably, or definitely related to a study procedure
- Death of a study subject

Safety outcomes as requested by regulatory agencies for each subject who has consented to this LTFU study and pregnancy outcome will also be collected during the follow-up period. Specific disorders will include **but are not limited to** incident cases of: systemic lupus erythematosus, rheumatoid arthritis, Guillain-Barre syndrome, acute disseminated encephalomyelitis and multiple sclerosis.

## 2.7 Statistical Analysis Plan Summary

In the absence of a control group in the LTFU study, it is not possible to perform analyses of efficacy. Instead, effectiveness will be measured in terms of incidence rates of disease, with analyses performed periodically until the end of the follow-up in Protocol 019-20. Three (3) analyses are planned, corresponding to the time when all participating subjects have completed each of the scheduled visits at Years 6, 8, and 10 relative to the base study Day 1. All subjects who received GARDASIL™ within the context of Protocols 019 base study or the 019-10 extension from the participating sites in Colombia will contribute to the effectiveness analyses. At each analysis time point, the cumulative and current (for the current interval) number of cases for each effectiveness endpoint will be counted by analytical cohort. Incidence rates based on accrued person-years of follow-up will be estimated together with a corresponding 95% confidence interval.

As an aid to interpretation, incidence rates observed during the LTFU study will be put in context with corresponding incidence rates observed during the base study to provide an assessment of vaccine effectiveness. If any other new data becomes available during the LTFU which provides relevant information for this study population, from Merck studies or the published literature, that will also be used to contextualize the incidence rates that will be observed during the LTFU study. All LTFU analyses will be specific to time since vaccination, so will be presented separately for Cohort 1 and Cohort 2. Cohort 1 includes subjects vaccinated with GARDASIL™ on Day 1 in the base study. Cohort 2 subjects were allocated to receive placebo during the base study and can be enrolled in the long term follow-up extension following receipt on GARDASIL™ in the first extension (Protocol 019-10).

Exploratory analyses may be done to assess the expected continued protection against recurrent infection in women who were seropositive and PCR negative to the relevant HPV type in the vaccine. Sensitivity analyses may be done to study expected *vs.* observed cases of disease due to recurrent HPV types.

The primary analysis approach for Cohort 1 will be per-protocol-efficacy (PPE), as defined during the base study. To be eligible for this population, subjects must (i) have received 3 doses of GARDASIL™ within 1 year, and (ii) have no protocol violations. Subjects will be considered cases related to a given HPV type provided the subject was negative to the respective HPV type by serology and PCR prior to vaccination (i.e., during the base study) and PCR negative through Month 7. For purposes of endpoint definition, only the Pathology Panel diagnosis will be considered. Supportive analyses will be done for the group naïve to the relevant HPV type (HNRT) and full analysis set (FAS). The HNRT population consists of all subjects who received at least 1 vaccination and were seronegative and PCR negative to the relevant type prior to vaccination. The FAS population includes all vaccinated subjects, regardless of HPV status. Subjects who were cases of the relevant endpoint during the main study will not be included in the LTFU analyses of that endpoint.

The base study PPE population definition can not be applied to Cohort 2 subjects due to absence of HPV serology and PCR status information on Cohort 2 subjects at the time immediately before injection of vaccine Dose 1, and absence of HPV PCR status at "Month 7", i.e., at 1-month post vaccine Dose 3. Therefore, the analysis on Cohort 2 subjects will be done on the FAS population.

The sample size for the effectiveness and immunogenicity components of the LTFU study is fixed by the number of Protocol 019 base study and 019-10 study participants from Colombia who are eligible and willing to participate in the LTFU study. There are approximately 800 subjects in each of Cohorts 1 and 2 in the participating sites in Protocol 019-20 who will contribute extension study data. Given this relatively small sample size, it is important to note that the expected number of endpoint cases that can potentially be observed over the LTFU period is low. For example, the incidence rate of HPV 16/18-related CIN2/3 in the per-protocol placebo group during the base study was 0.1 per 100 person-years. Table 2-1 illustrates incidence rate estimates and confidence intervals for the indicated numbers of cases that can potentially be observed during the LTFU period. It is clear that in many cases, the confidence intervals do not exclude the placebo incidence rate of 0.1.

Table 2-1

Example Incidence Rates for Numbers of Cases Observed During
Long-Term Follow-Up Period (Cohort 1 Subjects)

| Cumulative cases observed from start of LTFU | At 10 years from vaccination  Incidence/100 PY (95% CI) | |
| --- | --- | --- |
| 0 | 0 | (0.00, 0.08) |
| 1 | 0.02 | (0.00, 0.12) |
| 2 | 0.04 | (0.01, 0.15) |
| 3 | 0.06 | (0.01, 0.18) |
| Rate calculations assume full follow-up was obtained from 800 subjects in Cohort 1, to the indicated time points.  LTFU = Long Term Follow-up; PY = Patient years; CI = confidence interval | | |

# 3. PROTOCOL DETAILS

## 3.1 Rationale

### 3.1.1 Long Term Follow-Up

Results of the Phase III studies have demonstrated that GARDASIL™ is highly efficacious in preventing HPV 6-, HPV 11-, HPV 16-, and HPV 18-related CIN, AIS, cervical cancer (via CIN 2/3 and AIS), VIN, VaIN, condylomata acuminata, vulvar cancer (via VIN 2/3), and vaginal cancer (via VaIN 2/3) in the group that received GARDASIL™ compared to the placebo group in subjects who were not infected by the relevant HPV type at the start of the study. The base study was conducted to bridge to GARDASIL™ studies conducted in girls and women 9-26 years of age. The long term follow-up study is being conducted to assess safety, immunogenicity, and effectiveness in the population of women vaccinated at 24 to 50 years of age through 5 to 10 years following vaccine Dose 1.

## 3.2 Study Procedures

### 3.2.1 Concomitant Medication(s)/Treatment(s)

No study vaccinations will be administered during the LTFU study. If an investigator discovers that a subject received an additional dose of GARDASIL™ outside the context of the LTFU study, such discovery should be reported to the study sponsor.

### 3.2.2 Activity and Other Prerequisites

Subjects and study personnel should adhere to the following prerequisites and if necessary, any scheduled visit may be rescheduled within the allowed time range of ± 6 months of Year 6, 8, or 10 visits.

For study visits which include pelvic exams, study personnel should verify by verbal history that:

1. Subjects have refrained from douching/vaginal cleansing or using any vaginal medication or preparation for 48 hours prior to any study visit. If the subject has not refrained from douching or using a vaginal medication or preparation during the 48 hours prior to the scheduled or unscheduled visit, the exam and specimen collection must be delayed until the 48 hour requirement is met.
2. Subjects have refrained from sexual intercourse (including anal, vaginal, or genital/genital contact whether same sex or opposite sex) for 48 hours prior to any visit (scheduled or unscheduled), in an attempt to avoid detection of viral DNA which has been deposited in the vagina or on the perineal/perianal area during sexual intercourse and is not the result of ongoing infection. If the subject has not refrained from the specified sexual activity for 48 hours prior to the scheduled or unscheduled visit, the exam and specimen collection must be delayed until the 48 hour requirement is met.
3. Subjects have had no menses within 2 days of any study visit involving specimen collection. Any visit for specimen collection should be done at least 2 days after menses is completed. If possible, no specimen should be collected within 2 days prior to menses. If, despite the above, visible blood is noted in the vagina, then the specimen may be collected. The presence of visible blood in the vagina should be noted in the data collection module.

### 3.2.3 Procedures

The Study Flow Chart summarizes procedures for scheduled study visits and items appear in the order they should be performed. This section provides clarification on the scheduled study visit procedures (presented in the same order as on the Study Flow Chart).

#### 3.2.3.1 Informed Consent

Eligible subjects will be required to re-consent prior to initiating any procedures and subjects that decline will require documentation by the site regarding the refusal.

The investigator must obtain documented consent from each potential subject in biomedical research or when an investigational vaccine is administered to subject in a clinical study. If a Patient Package Insert (PPI) exists for any product being used in the study within the indication and dosage of the prescribing information, the consent form will reference the PPI and the investigator shall provide a copy of it to the subject for his/her review before the subject signs the Informed Consent Form. If the study is outside of the indication or dosage, the consent form shall include all relevant information from the PPI but the subject will not be given a copy of the PPI.

Consent must be documented by the subject dated signature on a Consent Form along with the dated signature of the person conducting the consent discussion.

If the subject is illiterate, an impartial witness should be present during the entire informed consent reading and discussion. Afterward, the subject should sign and date the informed consent, if capable. The impartial witness should also sign and date the informed consent along with the individual who read and discussed the informed consent (i.e., study staff personnel).

If the subject is legally incompetent (i.e., a minor or mentally incapacitated), the written consent of a parent, legal guardian or legal representative must be obtained. Depending on local law or review committee requirements such consent may also need to be signed by an impartial witness.

When the study population includes non-English speaking people, the information in the consent form should be translated and communicated to the subject in language understandable to the subject. Either the investigator or Merck may take the responsibility for the translation; however, documentation must exist to demonstrate who performed the translation and that the translation was verified by an individual other than the person who performed the translation. An accurately translated consent form should be provided with a written statement by the translator (whether the translator is the investigator, the Clinical Monitor, or a professional translator), indicating that the consent form is an accurate translation of the accompanying English version.

A copy of the signed and dated consent form should be given to the subject before participation in the trial.

The initial informed consent form and any subsequent revised written informed consent form, and any written information provided to the subject must receive the IRB/IEC’s approval/favorable opinion in advance of use. The subject or his/her legally acceptable representative should be informed in a timely manner if new information becomes available that may be relevant to the subject’s willingness to continue participation in the trial. The communication of this information will be provided and documented via a revised consent form or addendum to the original consent form.

A copy of the FDA Regulations Regarding Informed Consent and the World Medical Association Declaration of Helsinki are included in the administrative binder.

#### 3.2.3.2 Randomization/Allocation

No new randomization will be performed during the LTFU study. The allocation numbers assigned to study subjects from Colombia during the base study will be carried over to the LTFU study.

A single patient/subject cannot be assigned more than 1 allocation number.

#### 3.2.3.3 Treatment/Evaluation/Follow-Up Procedures

Inclusion/exclusion criteria will be reviewed prior to enrollment or any study procedures.

All subjects/patients will be given a card, identifying them as participants in a research study. The card will contain contact information (including direct telephone numbers) to be utilized in the event of an emergency.

No vaccination will be provided in the long term follow-up study.

A plastic, non lubricated single-use, individually wrapped plastic speculum will be used for gynecologic examinations.

Please refer to the Specimen Collection Procedures Manual for step-by-step specimen collection and handling procedures.

##### 3.2.3.3.1 Summary of Scheduled Study Visit Procedures

The following visit procedures will be performed at the Year 6, Year 8, and Year 10 visits:

1. Medical history
2. Gynecologic history
3. General physical exam
   - include weight, sitting blood pressure, sitting pulse, respirations, and oral temperature
   - check the heart, lungs, thyroid, skin, and breasts
4. External portion of wart/lesion inspection (vaginal inspection will occur after all specimens are collected)
5. Pelvic Exam with plastic speculum
6. Optional tests based on clinical findings (see section 3.2.3.3.4)
7. ThinPrep™ Pap test
8. Vaginal portion of wart/lesion inspection
9. Bimanual exams or palpation of uterus and adnexa

Perform bimanual exam only after all genital samples are collected to avoid possible contamination or bleeding that might affect the quality of the samples

1. Blood collection for anti-HPV serology test
2. Safety follow-up - See Section 3.4

##### 3.2.3.3.2 Procedures During Pregnancy and Lactation

All pregnancies are to be reported to the SPONSOR. If pregnancy develops the investigator will maintain regular contact with the subject for the purpose of pregnancy assessment, including a detailed interim medical history (medical procedures and medications taken during the pregnancy). The information is to be updated at least once each trimester. Pregnancies will be followed to the completion/termination of the pregnancy. If the pregnancy continues to term, the outcome (health of infant) must be reported to the SPONSOR. Information regarding this data management procedure will be provided by the SPONSOR.

The subject will complete the study visits as per protocol and at the investigator’s discretion. Collection of samples and performance of visits and/or procedures during pregnancy are up to the local standard of care and/or the investigator’s judgment. The use of a cytobrush (Cytobrush™) for ThinPrep™ Pap test collection is contraindicated in women that are 10 weeks pregnant or more, according to the current manufacturer’s label. The use of a cyto-broom (Wallach Papette™) is not contraindicated in pregnancy, according to the current manufacturer’s label. The use of a cyto-broom instead of Cytobrush™ will be allowed if the investigator decides to perform a ThinPrep™ Pap test during pregnancy. Routine study procedures that are contraindicated during pregnancy will not be performed and the visit will be postponed until the procedures can be performed.

Information on lactation will not be collected during Protocol 019-20.

##### 3.2.3.3.3 External Genital Inspection

An external genital inspection will be conducted to ascertain the presence of genital warts/lesions in the external genital regions. This inspection will be conducted prior to the pelvic exam. Symptomatic subjects (e.g., those experiencing pruritus, discomfort, presence of lumps or bumps) may be seen at a scheduled or unscheduled visit for further evaluation.

In addition to the external genital wart/lesion inspection, a vaginal wart/lesion inspection is conducted after collection of per-protocol specimens. Please refer to the Specimen Collection Procedures Manual for step-by-step genital wart/lesion specimen collection (includes equipment list) and handling procedures.

###### 3.2.3.3.3.1 Genital Wart/Lesion Treatment and the Event of Recurrence

Management of genital warts will be at the investigator’s discretion, per the site's standards and practice. Excision of the wart is the ***preferred*** study treatment. If excision is the treatment modality chosen by the investigator, all excised tissue is to be submitted to the central laboratory for analysis.

Treatment of genital warts by topical medications and cryotherapy are acceptable study procedures; however, these treatment modalities can be performed only after completing all biopsy requirements. A biopsy of the identified lesion(s) (e.g., morphology differs among identified lesions or more than one lesion identified) must be obtained prior to administering topical medications or cryotherapy. In addition, cryotherapy is to be performed only on the external genitalia (no cervical cryotherapy is allowed). Overall, the preferred method is to biopsy and treat at the same visit.

During genital wart treatment, follow-up biopsies should be obtained if new HPV-related lesions of differing morphology, and/or differing location appear. A recurrence of a genital wart/lesion is defined as the reappearance of a lesion of similar morphology in the same anatomical location within 2 months after complete resolution of the initial lesion. Recurring lesions will not be biopsied. Otherwise, all new lesions will be biopsied.

##### 3.2.3.3.4 Optional Tests for Clinical Symptoms and Clinical Abnormalities

###### 3.2.3.3.4.1 Optional Tests for Clinical Symptoms

Optional tests may be performed, if indicated and at the investigator's discretion, at any time during the study. Procedures for collection of certain optional tests may be located in the Specimen Collection Procedures Manual. Optional tests include:

- *Neisseria gonorrhoeae* culture
- *Chlamydia trachomatis* culture
- Syphilis detection test
- Hepatitis B surface antigen (HBsAG)
- Hepatitis C surface antigen (HCsAG)
- HIV antibody test
- Swab for Herpes Simplex Virus Culture
- Vaginal pH
- Wet mount for trichomonas and bacterial vaginosis
- Whiff test for bacterial vaginosis
- KOH test for yeast

Subjects who test positive for gonorrhea, Chlamydia, syphilis, HIV, hepatitis B, or hepatitis C should be referred for appropriate counseling and treatment. Treatment for Chlamydia or gonorrhea can be administered following specimen collection.

###### 3.2.3.3.4.2 Clinical Abnormalities

Any clinical abnormalities found at any time during the study will be referred for appropriate care:

- Vaginitis may be managed by the investigator according to the local standard of care only after visit procedures are performed.
- Cervicitis may be managed by the investigator. If there is clinical evidence of gross purulent cervicitis during a scheduled visit that involves a pelvic exam, the specimen collection should be postponed until after treatment or determination of nonspecific identifiable etiology.

##### 3.2.3.3.5 Pap Test for Liquid Cytology (ThinPrep™)

A ThinPrep™ Pap test for cytology will be performed at Year 6, Year 8, and Year 10 visits. All ThinPrep™ Pap tests will be tested at a cytology laboratory selected by the SPONSOR. Cytology specimens will be evaluated using the Bethesda System—2001. For all cytology diagnoses of Atypical Squamous Cells of Undetermined Significance (ASC-US), the SPONSOR central laboratory will automatically perform reflex HPV testing on residual ThinPrep™ material, using the Digene Hybrid Capture II™, High Risk/Low Risk Probes. If at least 1 probe is positive or if no result is obtained, the subject will be referred for colposcopy. If both probes are negative, the subject will return for Pap screening at the routine visit interval. Colposcopy is recommended for the cytology diagnosis of Atypical Squamous Cells, and cannot rule out high grade squamous epithelial lesions (ASC-H). All Pap reports should be reviewed and signed by an M.D. or D.O. investigator/subinvestigator.

Procedures should be performed for all subjects as per the protocol mandated instructions located in the SPONSOR’s Specimen Collection Procedures Manual. Specimens must also be shipped according to instructions summarized in the SPONSOR’s Specimen Collection Procedures Manual.

##### 3.2.3.3.6 Serum for Antibody Measurements

Cohort 1, the subjects vaccinated with GARDASIL™ on Day 1, will undergo anti-HPV serology testing at approximately 6, 8 and 10 years following Day 1 for anti-HPV 6, 11, 16, and 18 responses (see Section 3.3.3 for description of the cLIA assay used). Cohort 2, the subjects who were allocated to receive placebo during the base study, will be enrolled in the long term follow-up extension following receipt of GARDASIL™ in the first extension (Protocol 019-10). This cohort will have similar calendar time points for serological testing which correspond to approximately 1, 3, and 5 years following vaccination with GARDASIL™.

Please refer to the Specimen Collection Procedures Manual for step-by-step procedures for blood (serum) and retention serum collection and handling guidelines.

##### 3.2.3.3.7 Colposcopy/Biopsy/ Definitive Therapy Procedures

###### 3.2.3.3.7.1 Triage for Abnormal Pap Tests

Subjects with ThinPrep™ Pap tests obtained during the study showing abnormalities should be referred to colposcopy and biopsy according to the Mandatory Algorithm for Triage of Abnormal Pap tests to Colposcopy provided in Table 3-2. This is a protocol-prescribed strategy and any deviation will require prior approval by the SPONSOR. Colposcopy may also be performed in the case of an unresolved post-coital bleeding that persists after completion of appropriate work-up. Prior to a colposcopy for this indication, the case must be reviewed with the SPONSOR and a note to file must be generated.

If a subject is scheduled to undergo a hysterectomy, a colposcopy with biopsy should be performed.

Table 3-2

Mandatory Algorithm for Triage of Abnormal Pap Tests to Colposcopy

| ThinPrep™ Pap Result | Action |
| --- | --- |
| Negative for intraepithelial lesion or malignancy (includes reactive, reparative, inflammatory, etc.) | Routine visit interval as specified by the protocol. |
| Atypical Squamous Cells—Undetermined Significance (ASC-US) | Central laboratory performs reflex HPV testing on residual ThinPrep™ material (High Risk and Low Risk Probe, Hybrid Capture II, DIGENE™). If at least 1 probe is positive or if no result is obtained, the subject will be referred for colposcopy. If both probes are negative, then the subject will return for Pap screening at the routine visit interval. |
| Atypical Squamous Cells—cannot exclude HSIL (ASC-H) | Referral to colposcopy. |
| Low-grade Squamous Intraepithelial Lesion (LSIL) | Follow-up in 6 months |
| High-grade Squamous Intraepithelial Lesion (HSIL) | Referral to colposcopy. |
| Atypical Glandular Cells (to include atypical endocervical, endometrial, NOS; Adenocarcinoma in Situ, adenocarcinoma, etc.) | Referral to colposcopy. |
| Unsatisfactory | Repeat ThinPrep™ Pap test as soon as possible, but not less than 4 weeks from the Pap test that had the unsatisfactory finding. |
| Colposcopy should be performed according to the guidelines in this table or as indicated clinically over the ensuing period of follow-up.  Colposcopy must be performed within 2 months of the abnormal Pap result that required the referral to colposcopy. | |

###### 3.2.3.3.7.2 Colposcopy

All colposcopies will be performed at the study site. Colposcopy will be performed only by an experienced colposcopist (>50 colposcopies per year for at least 2 years). Subjects who undergo colposcopy may undergo biopsy if clinically indicated. Each discrete abnormal area that is observed on colposcopy should be biopsied. Separate biopsy forceps must be used for each of the discrete lesions that are biopsied.

To ensure that colposcopy practices and skill levels are standardized across investigator sites and colposcopists at all sites develop a uniform approach to all study colposcopies, colposcopists will be required to participate in Colposcopic Standardization Training. The purpose of this training is to evaluate the colposcopic practices at all investigator sites, to standardize colposcopy and biopsy practices, and to provide a guide for all aspects of the protocol related to colposcopy and histologic sampling of the cervix. An alternative to the SPONSOR organized colposcopy training, the “Comprehensive Colposcopy Course,” given by the American Society for Colposcopy and Cervical Pathology (ASCCP) will be accepted as equivalent for the purpose of standardization to participate in this study, provided it has been taken within a year of study startup.

If no lesion is noted on colposcopy, and the subject was referred based on a cytologic abnormality, endocervical curettage (ECC) may be performed if it is deemed by the investigator to be medically indicated. A curet may be used, but not a cytobrush, when obtaining the ECC specimen.

Please refer to Specimen Collection Procedures Manual for guidelines for colposcopic procedures.

###### 3.2.3.3.7.3 Procedures for Biopsy and Definitive Therapy

The following are general procedures for external genital biopsy, cervical biopsy, and definitive therapy that may be performed during the study. Specific step-by-step procedures can be found in the Specimen Collection Procedures Manual provided by the SPONSOR.

***Genital Wart/Lesion Biopsy***

Genital wart biopsy may be conducted at a scheduled or unscheduled visit. If a suspected lesion(s) (possibly, probably, or definitely HPV-related per the investigators judgment, or of unknown etiology) is identified in the vagina, vulvar, periurethral, perineal, and/or perianal region, a biopsy specimen is to be obtained and submitted to the central laboratory designated by the SPONSOR for review by a pathologist for the purpose of management of the subject. It is important to make an accurate evaluation of any identified lesions observed during the study on all study participants. After a thorough examination:

- The investigator should provide a clinical impression of the lesion, which should include 1 of the following: condyloma acuminata, a flat wart, a papular wart, a keratotic wart, other HPV-related lesion (e.g., VIN, Bowenoid papulosis, Bowen’s disease), or other non-HPV-related lesion.
- The location and anatomical area of each biopsied wart/lesion should be identified and noted (as per lab kit requisition supplied by the SPONSOR) and should also be recorded according to data management guidelines.
- Any suspicious area identified should be biopsied for evaluation.
- For multiple warts, select 1 external wart to biopsy that is most representative of the morphology and is most accessible.
- Additional biopsies may be performed either for warts in a different anatomical area and/or for warts that have a different clinical impression. For each biopsy, different instruments are to be used to prevent cross-contamination between specimens.
- Identified vaginal lesions (VaIN) are to be collected in a separate vaginal biopsy kit. Treatment for VaIN is per investigational site’s standards and practices.

Please refer to the Specimen Collection Procedures Manual for step-by-step genital wart/lesion biopsy collection and handling procedures.

Slides of wart/lesion biopsy or vaginal biopsy specimens will be prepared at the central pathology laboratory and reviewed by a pathologist for the purpose of management of the subject. The slides prepared from the genital wart/lesion biopsy (external and vaginal), as well as the investigator’s observation of whether the lesion is single or there are multiple lesions, will be submitted to the Independent Pathology Panel which will provide the final diagnosis for study purposes. The slides will be returned to the originating central pathology laboratory at the completion of review by the panel.

If the diagnosis of the Independent Pathology Panel is VIN 3/VaIN 3 or worse, and the diagnosis of the central laboratory is of lower grade abnormality, then the investigator will be notified of the discrepant diagnoses. The Independent Pathology Panel will be blinded to the results of the PCR analysis of the biopsy.

External genital and vaginal biopsy specimens will be sent to the SPONSOR's designee for HPV analysis. HPV analysis will be performed on Thinsection microtomy specimens. Each biopsy specimen will be analyzed by HPV PCR, regardless of whether an HPV-related histologic diagnosis is made, for the purpose of determining the causal HPV type in the lesion. HPV typing will be performed using Multiplex PCR Assay to detect vaccine HPV types. Samples that test negative for HPV vaccine types will be analyzed for non-vaccine HPV types using type-specific PCR for multiple high-risk HPV types. The interval between the genital wart/lesion biopsy and the next scheduled study visit that includes a complete gynecological examination must be ≥2 months.

Treatment for VIN, VaIN, and genital warts is per the investigational site’s standards and practices. During genital wart treatment, follow-up biopsies should be obtained if new HPV-related lesions of differing morphology, and/or differing location appear. A recurrence of a lesion is defined as the reappearance within 2 months of a lesion of similar morphology in the same anatomical location after complete resolution of the initial lesion. Recurring lesions will not be biopsied. Otherwise, all new lesions will be biopsied.

**A subject with an external genital lesion that is histologically confirmed to be HPV-related (e.g., VIN, VaIN, and genital warts) by the SPONSOR central laboratory is to be referred to colposcopy.**

***Cervical Biopsy and Endocervical Curettage***

If cervical biopsies and/or endocervical curettage (ECC) specimens are obtained, specimens must be sent to the central laboratory designated by the SPONSOR for analysis. This unscheduled visit(s) will be considered part of the study and any specimens collected will be handled in a protocol-specified manner.

Slides of cervical biopsy/ECC specimens will be prepared at the central laboratory selected by the SPONSOR and reviewed by a pathologist for the purpose of management of the subject. The slides will subsequently be reviewed by the Independent Pathology Panel for consensus diagnosis. The slides will be returned to the originating central pathology laboratory at the completion of review by the panel.

The investigator will be notified of any histologic diagnosis made by the Independent Pathology Panel that is more severe than the one made by the central laboratory pathologist. For the purposes of this protocol, the SPONSOR and the central laboratory have defined a discordant diagnosis as a case in which the Thin Prep™ Pap test is more severe than the subsequent cervical biopsy (and ECC, if obtained) diagnosis and the Pap test diagnosis would ultimately lead to definitive therapy. If the Pap diagnosis is AGC, AIS, ASC-H or LSIL, cannot exclude HSIL and the biopsy diagnosis is CIN 1 or less, then this is defined as discordant. The investigator will be notified of all discordant diagnoses. A discordant diagnosis will require a review of the results. If upon review the results are amended, the triage algorithm should be followed (if the Pap results are amended, i.e., not HSIL, not AGC, not ASC-H, then follow the routine visit schedule, or if the biopsy results are amended to show CIN 2/3 or worse, proceed to definitive therapy). If the discordant results are reviewed but not amended, a repeat Pap and a colposcopy/ECC are required (≥2 months after the last colposcopy/ECC). If the diagnosis of the pathology panel is CIN 2/3 or worse, and the diagnosis of the central laboratory is of a lower grade abnormality, then this is defined as a safety net case. Safety net notification does not require a review of the results.

If no lesion is noted on colposcopy and/or the colposcopy exam is unsatisfactory, and the subject was referred based on cytologic abnormality, an endocervical curettage (ECC) along with a careful inspection of the vagina may be performed if it is deemed by the investigator to be medically indicated. A curet may be used, but not a brush when obtaining the specimen. Thinsection HPV PCR analysis will be done on all ECC specimens, along with a histopathologic review. Additional Pap testing during the unscheduled colposcopy study visit is not allowed.

HPV analysis will be performed on biopsy tissue and Thinsection microtomy specimens. Each biopsy specimen will be analyzed by HPV PCR, regardless of whether an HPV-related histologic diagnosis is made, for the purpose of determining the causal HPV type in the lesion. HPV typing will be performed using Multiplex PCR Assay to detect vaccine HPV types on all samples. Samples will be analyzed for non-vaccine HPV types using Type-Specific PCR for High-Risk HPV Types, specifically HPV types HPV 31/33/35/39/45/51/52/56/58/59.

Subjects who undergo cervical biopsy during the study may remain in the study. The interval between the cervical biopsy and the next scheduled visit that includes a pelvic exam must be at least 2 months.

Please refer to the Specimen Collection Procedures Manual for step-by-step procedures for cervical biopsy and endocervical curettage collection and handling.

***Referral for Definitive Therapy***

Subjects who undergo cervical biopsy during the study will be referred for definitive therapy according to mandatory protocol prescribed guidelines including:

- Biopsy-confirmed CIN 2, CIN 3, or cervical cancer
- Two repeat HSIL diagnoses (regardless of whether they are consecutive or not) on ThinPrep™ Pap test without confirmed CIN 2, CIN 3, or cervical cancer on biopsy or ECC
- An ECC demonstrating CIN 2, CIN 3, AIS, or cervical cancer
- A single HSIL Pap with an unsatisfactory colposcopic examination including a negative examination of the vagina and a negative ECC
- Atypical Glandular Cells or AIS on Pap result with unsatisfactory colposcopy or negative biopsy or ECC; or

Flow charts illustrating these indications for definitive therapy is provided in Section 7 (Attachments).

Cervical tissue specimens will be submitted to the central laboratory selected by the SPONSOR for histopathologic review. In addition, specimens will be prepared by the central lab for HPV PCR analysis by the SPONSOR or its designee using the Multiplex PCR Assay to detect vaccine and non-vaccine HPV types on all samples.

**Acceptable Study Procedures for Definitive Therapy**

- LEEP (Loop Electrosurgical Excision Procedure) is the preferred method for definitive therapy.
- The deeper top hat LEEP cone is also acceptable.
- Laser conization is an acceptable study procedure if LEEP is not the standard practice of care.
- Cold-knife conization and ablative therapy (cryotherapy, laser therapy) should be reserved only for rare instances in which definitive therapy is required and LEEP is not indicated. Cold-knife conization will be considered a study procedure. Biopsy specimens are to be obtained from the areas of highest abnormalities at the time of the conization procedure. This is to be sent to the SPONSOR for histopathologic review and HPV analysis.

The LEEP is a shallow pass to remove the cervical transformation zone. It may be a single or multiple pass and is ~8 mm deep. The top hat cone implies a deeper wedge resection, ~15 to 25 mm deep. It is performed by the “top-hat” method of successively more internal and smaller loops.

Laser Conization excises tissue by laser beam, using small depressor instruments or hooks to position the cone for optimal excision. LEEP and Laser conization are study procedures.

LEEP excisional procedures are contraindicated in the following conditions: allergy to all local anesthetics; pregnancy; severe acute cervicitis (severe acute cervicitis should be diagnosed and treated, and excision conducted after the infection has resolved); obvious invasive cancer; significant glandular neoplasia (AGUS favor neoplasia, AIS, Adenocarcinoma); and microinvasive cancer.

The LEEP provider should be an experienced physician (defined as performing 20 LEEP procedures per year for at least 2 years) and must have documented formal instruction, i.e., residency or postgraduate course or American Society for Colposcopy and Cervical Pathology (ASCCP) training course.

Subjects who undergo definitive therapy (LEEP, laser conization, and cold-knife conization) will continue to be followed through completion of scheduled visits. The interval between definitive therapy and the next scheduled visit which includes a pelvic exam must be at least 2 months. If the interval is <2 months, the next visit should be postponed. Follow-up care post-definitive therapy (LEEP, conization, laser conization) should occur as per the investigator sites’ standards and practices.

**Nonstudy Procedure for Definitive Therapy and Hysterectomy**

Cervical ablative therapy (cervical cryotherapy, laser therapy) and hysterectomy are not study procedures. If a subject undergoes cervical ablative therapy (cervical cryotherapy, or cervical laser therapy) or hysterectomy during the study, biopsy specimens should be obtained, under colposcopic conditions, from areas of highest abnormalities prior to the procedure and sent to the SPONSOR's designee for histopathologic review and HPV analysis.

Subjects who undergo cryotherapy or other ablative therapy will continue to be followed through completion of scheduled visits; however, they will not be further eligible for the cervical efficacy endpoint. The interval between definitive therapy and the next scheduled visit that includes a pelvic exam must be at least 2 months. If the interval is <2 months, the next visit should be postponed.

**Procedure for LEEP**

Note: The LEEP should be done under colposcopic control. All instruments used for the procedure must be nonconductive.

A biopsy of the affected tissue should be obtained immediately prior to this procedure. Please refer to the Specimen Collection Procedures Manual for step-by-step LEEP collection and handling procedures.

**Procedure for Laser Conization**

The laser conization provider should be an experienced physician (defined as performing at least 20 laser conization procedures per year for at least 2 years) and must have documented formal instruction, i.e., residency or postgraduate course or American Society for Colposcopy and Cervical Pathology (ASCCP) training course. Similar requirements for countries outside the United States apply.

A biopsy of the affected tissue should be obtained immediately prior to this procedure. Please refer to the Specimen Collection Procedures Manual for step-by-step laser conization collection and handling procedures.

**Cold-Knife Conization**

Cervical cold-knife conization is a study procedure but should be performed only when the LEEP procedure is not indicated. A cervical cold-knife conization specimen represents a conically shaped section of the cervix that will vary in size according to the lesion. A broad, shallow conization would be performed for a predominantly exocervical lesion and a narrow, deep conization is appropriate for a predominantly endocervical lesion. A biopsy of the affected tissue should be obtained immediately prior to this procedure and the specimen should be oriented and submitted to the central laboratory as described the Specimen Collection and Procedures Manual.

If a subject undergoes cervical ablative therapy (cervical cryotherapy, or cervical laser therapy) or a hysterectomy during the study, biopsy specimens obtained at the time of the procedure will be sent to the SPONSOR’s central laboratory for histopathologic review and HPV analysis. Ablative therapy and hysterectomy are not study procedures.

**Procedures Outside the Context of the Study**

Pap, colposcopy, biopsy, and definitive therapy outside the study should be strongly discouraged. If a subject does undergo a study procedure outside of the study, all efforts will be made to obtain any: (1) Pap, colposcopy, or operative report; (2) diagnostic slides; (3) local pathology diagnosis; and (4) tissue block for diagnostic slide preparation and HPV analysis. (See Section 7 (Attachments), “Procedure for Handling Biopsies Collected Out of Context of Study [BOCS] in the Merck HPV Program,” and “Procedure for Handling Pap Tests Collected Out of Context of Study [POCS] in the Merck HPV Program.”)

#### 3.2.3.4 Blinding/Unblinding

All subjects in this study will have been vaccinated with GARDASIL™ within the context of either Protocol 019 base study or 019-10 extension prior to enrollment in the LTFU study. The study subjects, study site investigators and personnel, and Merck personnel (including clinical, statistics, and regulatory) are all aware that participants in this LTFU study have been vaccinated with GARDASIL™. Therefore, treatment assignment blinding of study subjects and personnel involved in the conduct of this LTFU is not applicable to this study.

#### 3.2.3.5 Discontinuation/Withdrawal from Study

Subjects/patients may withdraw at any time or be dropped from the study at the discretion of the investigator should any untoward effects occur. In addition, a subject/patient may be withdrawn by the investigator or the SPONSOR if he/she violates the study plan or for administrative and/or other safety reasons. The investigator or study coordinator must notify the SPONSOR immediately when a subject/patient has been discontinued/withdrawn due to an adverse experience (telephone or FAX). When a subject/ patient discontinues/withdraws prior to study completion, all applicable activities scheduled for the final study visit should be performed at the time of discontinuation. Any adverse experiences which are present at the time of discontinuation/withdrawal should be followed in accordance with the safety requirements outlined in section 3.4 SAFETY MEASUREMENTS - DETAILS.

### 3.2.4 STUDY DURATION AND SUBMISSION OF DATA

For each subject enrolled, the duration of the study may be as long as 10 years (relative to the time of injection of vaccine Dose 1). If a subject requests to discontinue/withdraw prior to the end of the LTFU study, the subject should be asked to return for a final visit if it has been at least 1 year since the last visit that included a pelvic exam. The visit should not be done if it is medically contraindicated or subject refuses. This visit would consist of the same exams, specimen collection, and tests conducted at the Year 10 visit. The subject would be discontinued from the study at the end of this final visit.

#### 3.2.4.1 Subject Retention and Relocation

Study retention activities will be performed, including ensuring that the study site has updated addresses, telephone numbers, or other contact information for the subject.

There will be no subject relocation during the long term follow-up extension.

## 3.3 Efficacy/Pharmacokinetic/Immunogenicity, etc. Measurements

### 3.3.1 Definition of the Effectiveness and HPV Type Replacement Endpoints

Effectiveness and HPV type replacement endpoints are as defined in Sections 2.5.2 and 2.5.3 of this protocol.

### 3.3.2 Blinding of HPV Results

As stated in Section 2.7, three analyses are planned corresponding to the time when all participating subjects have completed each of the scheduled visits at Years 6, 8, and 10 relative to the base study Day 1.

At Years 6 and 8: After all subjects have completed their study visit at each of Years 6 and 8, the study data will be checked for completeness and will be frozen for the purpose of conducting two of the three planned analyses. Merck personnel (including clinical, statistics, and regulatory) will conduct the analysis, write a report, and communicate the results to regulatory agencies and study site investigators. Therefore, Merck personnel at this time will be unblinded to both subject-level and aggregate cohort-specific results of Merck’s type-specific HPV PCR assay (both on routine visits and on biopsy Thinsections). The study subjects, site investigators and personnel, laboratory personnel, and Independent Pathology Panel members at this time will remain blinded to subject-level results of Merck’s type-specific HPV PCR assay. Study site investigators who will be provided a copy of the Year 6 and the Year 8 report will be unblinded to aggregate, cohort-level type-specific HPV PCR assay results.

At Year 10: After all subjects have completed the Year 10 study visit, the study data will be checked for completeness and will be frozen for the purpose of conducting the third of the three planned analyses. Merck personnel will conduct the analysis, write a report, and communicate the results to regulatory agencies and study site investigators. Therefore, Merck personnel at this time will be unblinded to both subject-level and aggregate cohort-specific results of Merck’s type-specific HPV PCR assay. The study subjects, site investigators and personnel, laboratory personnel, Independent Pathology Panel members at this time need not remain blinded to subject-level results of Merck’s type-specific HPV PCR assay and may be unblinded if necessary. Study site investigators who will be provided a copy of the Year 10 report will be unblinded to aggregate, cohort-level type-specific HPV PCR assay results.

### 3.3.3 Immunogenicity

**HPV-Luminex Assay (cLIA)**

All subjects will undergo anti-HPV serology testing for anti-HPV 6, 11, 16, and 18 responses (using competitive immunoassays developed by Merck Research Laboratories using technology from the Luminex Corporation, Austin, TX). The purpose of the quadrivalent human papillomavirus (HPV)-Luminex assay is to detect antibody to HPV virus-like particles (VLPs), serotypes 6, 11, 16, 18 before and after vaccination with GARDASIL™. This is the primary assay used by PPD Vaccines and Biologics (formerly the Virus and Cell Biology serology laboratory of Merck Research Laboratories (MRL)) to evaluate the serological response to the vaccine. Yeast-derived VLPs are coupled to a set of 4 distinct fluorescent Luminex microspheres. Antibody titers are determined in a competitive format, where known type-specific phycoerythrin (PE)-labeled neutralizing monoclonal antibodies (mAbs) compete with the subject’s serum antibodies for binding to conformationally sensitive neutralizing epitopes on the VLPs. The fluorescent signals from the bound HPV-specific detection mAbs are inversely proportional to the subject’s neutralizing antibody titers. Fluorescent value readings for clinical samples are referenced against a monkey reference serum standard curve and the concentrations of anti-HPV present are reported in milli-Merck Units per milliliter (mMU/mL).

A fixed cutoff will be used in the assays. The cutoff was derived by repeatedly testing a panel of positive and negative samples against the standard curve. Any sample with a value less than the cutoffs will be considered serostatus negative. Samples with values equal to or greater than the cutoff will be considered serostatus positive. Samples are read from a standard curve, corrected for dilution as needed, and reported in milli-Merck Units per milliliter (mMU/mL). The cutoffs for the HPV-6, -11, -16, and -18 competitive Luminex immunoassay are 20 mMU/mL, 16 mMU/mL, 20 mMU/mL, and 24 mMU/mL, respectively.

All serological testing in Protocol 019 is being done by LUMINEX II.

### 3.3.4 PCR Assays to Detect HPV in Clinical Specimens

All TS microtomy biopsy specimens will be tested for detection of HPV types 6, 11, 16, 18, 31, 33, 35, 39, 45, 51, 52, 56, 58, and 59. HPV types 6, 11, 16, 18, 31, 33, 45, 52, and 58 will be analyzed by type-specific multiplex (L1, E6, E7 gene detection) PCR assay (described in Section 3.3.4.1). HPV types 35, 39, 51, 56, and 59 will be analyzed by the duplex (E6, E7 gene detection) PCR assay (using the preparation method described in Section 3.3.4.1).

Upon HPV type identification of the biopsy thin-sections, and case assignment via positive consensus of histology diagnosis and HPV PCR, the cases will be reviewed for HPV type mixed-infections which will be noted. Exploratory studies of these HPV type mixed-infection tissues may be conducted. Additional thin sections will be required and adjudication of the proximal sections by thin section HPV multiplex PCR will be conducted. Briefly, additional 4-5 micrometer sections of the mixed-infection tissue paraffin blocks will be cut using a fresh disposable blade per block and applying the standard procedures implemented to ensure no contamination in thin sections processing for PCR. Seven sections will be cut. The first two and last two will be individually placed in tubes (tube 1, 2, 6 and 7) and tested by HPV Multiplex PCR to confirm the mixed-type status in the book-end sections of this second, deeper cut in the paraffin tissue block. The middle three sections will be applied to membrane-coated slides with diluted tissue adhesive (TA, Diagnostic Products Corp.) and stained with hematoxylin and eosin (HE). The margins of the cancerous cell populations will be microscopically defined by the pathologists, photographed and Laser Microdissection of Cells (LMC) performed [3]. The dissected cell clusters will be collected, DNA extracted and HPV Multiplex PCR tested. All three sections 3, 4 and 5 will be individually analyzed and tested.

#### 3.3.4.1 Multiplex PCR Assays

The following procedures will be done at PPD Vaccines and Biologics, Wayne, PA or a central laboratory designated by SPONSOR. For the detection of HPV Types 6, 11, 16, 18, Thinsection microtomy specimens are received and then prepared for multiplex PCR using a DNA purification method (Qiagen Technology Kit). Multiplex PCR (based on real-time fluorescent PCR) allows the simultaneous detection of 3 gene products (L1, E6, and E7) for a given HPV type in 1 reaction. The HPV type-specific primer pairs based on the published HPV L1, E6, and E7 sequences, are used to specifically amplify a portion of each gene simultaneously. The specific amplicons are detected in real-time by fluorescently-labeled oligonucleotide probes. The gene-specific oligonucleotide probes are each labeled with a different fluorescent label and the fluorescent emission is captured during PCR cycling by the real-time PCR instrument.

After analysis of the raw fluorescent data by the real-time PCR instrument's software, a threshold cycle (Ct), which represents the PCR cycle at which an increase in reporter fluorescence above a baseline signal can first be detected, is determined. Each gene-specific assay (i.e., gene-specific dye layer) is considered positive if the Ct is <45 cycles. A gene-specific assay is considered negative if the Ct = 45. A sample is called positive when 2 or 3 genes are positive (Ct <45 cycles) or when the same single gene scores positive (Ct <45 cycles) on consecutive tests.

The multiplex or duplex PCR assay will also be run to detect additional HPV Types including 31, 33, 35, 39, 45, 51, 52, 56, 58, and 59.

#### 3.3.4.2 Quantitative PCR Assays

The following procedures will be done at a central laboratory designated by the SPONSOR: Tissue samples are prepared for PCR using a DNA purification method (Qiagen Technology Kit)**.** Samples previously determined to be positive by multiplex PCR (see above) may be further analyzed by HPV type-specific primers based on the HPV E7 genes only (see above) to determine the HPV genome copy number. Copy number determination is by standard curve using qualified plasmid standards for the HPV E7 genes.

### 3.3.5 PCR Assays to Detect HPV in Thinsections of Biopsy Tissue

PCR assays to determine HPV in biopsy Thinsections will be performed at a central laboratory designated by the SPONSOR. Thinsections are prepared for PCR using a DNA purification method (Qiagen Technology Kit). HPV 6, 11, 16, and 18 Multiplex PCR (see Section 3.3.4.1) will then be performed on a fraction of the DNA prepared from each Thinsection to determine the HPV type in the Thinsection. Thinsections positive by HPV multiplex PCR for HPV 6, 11, 16, and 18 may be further analyzed by HPV type-specific primers based on the HPV E7 genes only to quantify viral load (see above). Thinsections will also be further analyzed by type-specific multiplex or duplex PCR assays for additional HPV Types including 31, 33, 35, 39, 45, 51, 52, 56, 58, and 59. For description of these assays, see Section 3.3.4.1.

### 3.3.6 Preparation and Disposition of Thinsections of Biopsy Tissue

The following procedures will be performed at a central laboratory designated by the SPONSOR. The procedures will be performed by an experienced, qualified histotechnologist according to the central laboratory's SOP. The histotechnologist will assure that the microtome and work areas are clean and free of contaminants. All Thinsection microtomy for PCR will be performed at a time when all other routine work has been completed, so that potential contaminations can be minimized. Prior to sectioning each block, a new blade will be installed in the microtome. The blade will only be positioned so that it is at the left margin of the blade surface. Technicians sectioning study blocks will utilize “biologically clean” gloves while handling the blocks (new gloves for each block). First, the histotechnologist will face the block by removing two 4‑micron sections from the face of the block. These sections will be discarded. Using sterile plastic forceps, the next two 4‑micron paraffin sections are collected and floated in a water bath for the preparation of 1 H&E slide (Slide 1, with 2 sections).

There will be 9 individual tubes (Tube 1, 2, 3, 4, 5, 6, 7, 8, 9), each containing one 4‑micron section. The pair of sterile plastic forceps used each time is then discarded after placing the cut section in each tube. Each tube is then placed inside a plastic sleeve and sealed.

Two additional 4-micron sections are cut and 2 sections each are placed on 1 slide (Slide 2 with 2 sections each) for H&E staining. All H&E slides (Slides 1 and 2) will be have a histopathologic review by the central laboratory's pathologist.

Slides and tubes should be labeled with subject's allocation number. The specimen tubes are collated with the appropriate specimen requisition and prepared for shipping to Covance Central Laboratory and then in turn, shipped on to MRL.

The microtome is cleaned in preparation for the next block and the process above is repeated. The microtome blade is replaced with a new blade and adjusted for each new biopsy block and the same procedure is to be followed. A new pair of clean gloves and a new pair of clean, disposable forceps will be used for each block being sectioned. The “used” blade may be retained for cutting non-PCR blocks. The total number of sections to be cut from each block is 13. A total of 2 slides and 9 tubes:

Slide 1 (H&E), with 2 sections each, stained.

Tubes 1, 2, 3, 4, 5, 6, 7, 8, 9 (HPV PCR Analysis), 1 section per tube.

Slide 2 (H&E), with 2 sections each, stained.

### 3.3.7 Conduct of the Clinical Trial

The trial will be conducted under the scrutiny of a Steering Committee. The clinical efficacy endpoints will be adjudicated by the Independent Pathology Panel, responsible for the definitive pathologic diagnoses in all clinical conditions which may be considered as possible endpoints in subjects in this trial. The operation of the Steering Committee and the Independent Pathology Panel are described below.

#### 3.3.7.1 Responsibility of the Steering Committee

The Steering Committee consists of specialists in the field of gynecology, oncology, virology, pathology, epidemiology, and biostatistics. The Steering Committee provides the overall scientific and operational direction for the trial through consideration of recommendations from its working committees. The Steering Committee is responsible for the conduct of the trial according to the highest scientific and ethical standards, as well as approving revisions and amendments to the protocol. The Steering Committee will remain blinded to results during the course of the study. The Steering Committee must approve all scientific reports for publication concerning the main findings of the trial. The Steering Committee will meet at least twice during the LTFU study. A Publications Subcommittee will be appointed.

Membership of the Steering Committee is composed of representatives from the participating country in the clinical trial, MRL Clinical Monitors, Clinical Research Specialist, and the Blinded Program Statistician.

#### 3.3.7.2 Adjudication Procedures

The Independent Pathology Panel consisting of up to 5 pathologists, will be responsible for providing the definitive pathologic diagnoses of cervical and vaginal biopsies, external genital lesion biopsies, endocervical curettage, and definitive therapy specimens for the purpose of determining the presence of endpoints in the study. This is the same Independent Pathology Panel used in the base protocol. The SPONSOR and the Independent Pathology Panel will follow established guidelines for review of the slides. The panel will prepare reports on each possible event without information on vaccine allocation that might identify the subject’s study treatment.

## 3.4 Safety Measurements

### 3.4.1 Clinical and Laboratory Measurements for Safety

Eligible subjects who enroll in the long term follow-up after receiving GARDASIL™ vaccination series will be followed for (1) vaccine- or study procedure-related serious adverse experiences and (2) serious adverse experiences resulting in death (3) pre-specified medical conditions including, **but are not limited to,** incident cases of: systemic lupus erythematosus, rheumatoid arthritis, Guillain-Barre syndrome, acute disseminated encephalomyelitis and multiple sclerosis, and (4) pregnancy follow-up outcomes.

### 3.4.2 Recording Adverse Experiences

An adverse experience is defined as any unfavorable and unintended change in the structure, function, or chemistry of the body temporally associated with the use of the SPONSOR’s product, whether or not considered related to the use of the product. Any worsening (i.e., any clinically significant adverse change in frequency and/or intensity) of a preexisting condition which is temporally associated with the use of the SPONSOR’s product, is also an adverse experience.

Changes resulting from normal growth and development which do not vary significantly in frequency or severity from expected levels are not to be considered adverse experiences. Examples of this may include, but are not limited to, teething, typical crying in infants and children, and onset of menses or menopause occurring at a physiologically appropriate time.

The following safety measurements will be collected from the time the consent form is signed through the completion of the year-10 visit:

Serious Adverse Experiences (SAEs) (as defined in the detailed protocol) judged by the study investigator to be possibly, probably, or definitely related to prior administration of GARDASIL; Serious Adverse Experiences (as defined in the detailed protocol) judged by the study investigator to be possibly, probably, or definitely related to a study procedure; and Death of a study subject.

### 3.4.3 Definition of an Overdose for This Protocol

No vaccination will be provided in the long term follow-up and therefore no definition of overdose is associated with this protocol. However, if an investigator discovers that a subject received an additional dose of GARDASIL™ outside the context of the study, this should be reported to the sponsor.

#### 3.4.3.1 Reporting of Overdose to SPONSOR

If an adverse experience(s) is associated with (“results from”) the overdose of test drug or vaccine, the adverse experience(s) is reported as a serious adverse experience, even if no other criteria for serious are met.

If a dose of test drug or vaccine meeting the protocol definition of overdose is taken without any associated clinical symptoms or abnormal laboratory results, the overdose is reported as a non-serious Event of Clinical Interest (ECI), using the terminology “accidental or intentional overdose without adverse effect.”

All reports of overdose with and without an adverse experience must be reported within 24 hours to one of the individuals listed on the sponsor contact information page found in the Administrative Binder.

### 3.4.4 Reporting of Pregnancy to SPONSOR

Although not considered an adverse experience, it is the responsibility of investigators or their designees to report any pregnancy in a subject/patient (spontaneously reported to them) which occurs during the study or within 14 days of completing the study. All subjects/patients who become pregnant must be followed to the completion/termination of the pregnancy. If the pregnancy continues to term, the outcome (health of infant) must also be reported to one of the individuals listed on the SPONSOR Contact Information page found in the Administrative Binder.

### 3.4.5 Immediate Reporting of Adverse Experiences to the SPONSOR

#### 3.4.5.1 Serious Adverse Experiences

Serious adverse experiences that are considered by the investigator to be possibly, probably, or definitely related to a previous vaccination of GARDASIL, or serious adverse experiences considered by the investigator to be related to a study procedure, or death due to any cause, which occurs to any subject from the time the consent is signed through the 10-year final visit, must be reported within 24 hours to one of the individual(s) listed on the sponsor contact information page found in the Administrative Binder.

Additionally, any serious adverse experience brought to the attention of an investigator who is a qualified physician at any time outside of the time period specified in the previous paragraph also must be reported immediately to one of the individuals listed on the sponsor contact information page (found in the administrative binder) if the event is either:

1. A death which resulted in the subject/patient discontinuing the study

or

2. A serious adverse experience that is considered by an investigator who is a qualified physician to be possibly, probably, or definitely vaccine related.

3. A serious adverse experience that is considered by an investigator who is a qualified physician to be possibly, probably, or definitely protocol-specified procedure related.

All subjects/patients with serious adverse experiences must be followed up for outcome.

All subjects who become pregnant must be followed to the completion/termination of the pregnancy.

### 3.4.6 Evaluating Adverse Experiences

Refer to Table 3-3 for instructions in evaluating adverse experiences.

Table 3-3

An investigator who is a qualified physician, will evaluate all adverse experiences as to:

| **Maximum** | **Mild** | awareness of sign or symptom, but easily tolerated (for pediatric studies, awareness of symptom, but easily tolerated) |
| --- | --- | --- |
| **Intensity** | **Moderate** | discomfort enough to cause interference with usual activity (for pediatric studies, definitely acting like something is wrong) |
|  | **Severe** | incapacitating with inability to work or do usual activity (for pediatric studies, extremely distressed or unable to do usual activities) **Injection site redness or swelling from the day of vaccination through Day 4 post-vacc will be evaluated by maximum size.** |
| **Seriousness** | A serious adverse experience is any adverse experience occurring at any dose that: | |
|  | **†Results in death; or** | |
|  | †**Is life threatening; or** places the subject/patient, in the view of the investigator, at immediate risk of death from the experience as it occurred [Note: This does not include an adverse experience that, had it occurred in a more severe form, might have caused death.]; or | |
|  | **†Results in a persistent or significant disability/incapacity** (substantial disruption of one’s ability to conduct normal life functions); or | |
|  | †**Results in or prolongs an existing inpatient hospitalization** (hospitalization is defined as an inpatient admission, regardless of length of stay, even if the hospitalization is a precautionary measure for continued observation.) (Note: Hospitalization [including hospitalization for an elective procedure] for a preexisting condition which has not worsened does not constitute a serious adverse experience.); or | |
|  | †**Is a congenital anomaly/birth defect** (in offspring of subject/patient taking the product regardless of time to diagnosis); or | |
|  | **Is a cancer; or** | |
|  | **Is an overdose** (Whether accidental or intentional.) Any overdose whether or not associated with an adverse experience must be reported within 24 hours. | |
|  | **Other important medical events** that may not result in death, not be life threatening, or not require hospitalization may be considered a serious adverse experience when, based upon appropriate medical judgment, the event may jeopardize the subject/patient and may require medical or surgical intervention to prevent one of the outcomes listed previously (designated above by a †). | |
| **Duration** | Record the start and stop dates of the adverse experience. If less than 1 day, indicate the appropriate length of time and units | |
| **Action taken** | Did the adverse experience cause the test vaccine to be discontinued? | |
| **Relationship to test vaccine** | Did the test vaccine cause the adverse experience? The determination of the likelihood that the test vaccine caused the adverse experience will be provided by an investigator who is a qualified physician. The investigator’s signed/dated initials on the source document or worksheet, that supports the causality noted on the AE form, ensures that a medically qualified assessment of causality was done. This initialed document must be retained for the required regulatory time frame. The criteria below are intended as reference guidelines to assist the investigator in assessing the likelihood of a relationship between the test vaccine and the adverse experience based upon the available information.  **The following components are to be used to assess the relationship between the test vaccine and the AE;** the greater the correlation with the components and their respective elements (in number and/or intensity), the more likely the test vaccine caused the adverse experience (AE): | |
|  | **Exposure** | Is there evidence that the subject/patient was actually exposed to the test vaccine such as: reliable history, acceptable compliance assessment (e.g. diary), seroconversion or identification of vaccine virus in bodily specimen? |
|  | **Time Course** | Did the AE follow in a reasonable temporal sequence from administration of the test vaccine?  Is the time of onset of the AE compatible with a vaccine-induced effect? |
|  | **Likely Cause** | Is the AE not reasonably explained by another etiology such as underlying disease, other drug(s)/vaccine(s), or other host or environmental factors |
| **Relationship** | **The following components are to be used to assess the relationship between the test vaccine and the AE: (continued)** | |
| **to test vaccine** | **Dechallenge** | (not applicable for vaccines) |
| **(continued)** | **Rechallenge** | Was the subject/patient reexposed to the test vaccine in this study?  If yes, did the AE recur or worsen?  If yes, this is a positive rechallenge. If no, this is a negative rechallenge.  (Note: This criterion is not applicable if: (1) the initial AE resulted in death or permanent disability, or (2) the study is a single-dose vaccine study.) NOTE: IF A RECHALLENGE IS PLANNED FOR AN ADVERSE EVENT WHICH WAS SERIOUS AND WHICH MAY HAVE BEEN CAUSED BY THE TEST VACCINE, OR IF REEXPOSURE TO THE TEST VACCINE POSES ADDITIONAL POTENTIAL SIGNIFICANT RISK TO THE SUBJECT/PATIENT, THEN THE RECHALLENGE MUST BE APPROVED IN ADVANCE BY THE U.S. CLINICAL MONITOR AND THE INSTITUTIONAL REVIEW BOARD/INDEPENDENT ETHICS COMMITTEE. |
|  | **Consistency with Study Vaccine Profile** | Is the clinical/pathological presentation of the AE consistent with previous knowledge regarding the test vaccine or vaccine class pharmacology or toxicology? |
| The assessment of relationship will be reported on the case report forms /worksheets by an investigator who is a qualified physician according to his/her best clinical judgment, including consideration of the above elements. | | |
| **Record one of the following:** | | **Use the following scale of criteria as guidance (not all criteria must be present to be indicative of a vaccine relationship).** |
| **Yes, there is a reasonable possibility of vaccine relationship.** | | There is evidence of exposure to the test vaccine. The temporal sequence of the AE onset relative to the administration of the test vaccine is reasonable. The AE is more likely explained by the test vaccine than by another cause.  **Depending on data collection method employed, vaccine relationship may be further graded as follows:** |
|  | **Definitely related** | There is evidence of exposure to the test vaccine. The temporal sequence of the AE onset relative to administration of the test vaccine is reasonable. The AE is more likely explained by the test vaccine than by another cause. Dechallenge is positive. Rechallenge (if feasible) is positive. The AE shows a pattern consistent with previous knowledge of the test vaccine or test vaccine class. |
|  | **Probably related** | There is evidence of exposure to the test vaccine. The temporal sequence of the AE onset relative to administration of the test vaccine is reasonable. The AE is more likely explained by the test vaccine than by another cause. Dechallenge (if performed) is positive. |
|  | **Possibly related** | There is evidence of exposure to the test vaccine. The temporal sequence of the AE onset relative to administration of the test vaccine is reasonable. The AE could have been due to another equally likely cause. Dechallenge (if performed) is positive. |
| **No, there is not a reasonable possibility of vaccine relationship** | | Subject did not receive the test vaccine OR temporal sequence of the AE onset relative to administration of the test vaccine is not reasonable OR there is another obvious cause of the AE. (Also entered for a subject with overdose without an associated AE.)  **Depending on data collection method employed, vaccine relationship may be further graded as follows:** |
|  | **Probably not related** | There is evidence of exposure to the test vaccine. There is another more likely cause of the AE. Dechallenge (if performed) is negative or ambiguous. Rechallenge (if performed) is negative or ambiguous. |
|  | **Definitely not related** | The subject/patient did not receive the test vaccine. **OR** Temporal sequence of the AE onset relative to administration of the test vaccine is not reasonable. OR There is another obvious cause of the AE. |

### 3.4.7 SPONSOR Responsibility for Reporting Adverse Experiences

All adverse experiences collected via study guidelines will be reported to regulatory agencies, IRB/IECs, and investigators in accordance with all applicable global laws and regulations.

The investigator is responsible for obtaining Review Board approval of the protocol, as well as approval of all subsequent major changes, in compliance with local law. Copies of these approvals must be forwarded to the SPONSOR. The IRB will comply with all federal, state, and local laws. Particular attention is drawn to the Food and Drug Administration Regulations for Institutional Review Boards (21 CFR, Part 56), and the International Conference on Harmonisation (ICH) Guidelines for Good Clinical Practices for IRB/IEC Committees. Copies of relevant information derived from these guidelines are included in the administrative binder. The investigator is responsible for obtaining initial and continuing review (at intervals not less than once per year) of the study by an IRB. Written approval from the IRB must be forwarded to the SPONSOR before clinical supplies will be shipped. For continuing studies, written approval from the IRB must be sent to the SPONSOR at intervals not to exceed 1 year.

The investigator shall also obtain from the IRB and submit to the SPONSOR, a signed statement indicating that it complies with Good Clinical Practices. A sample IRB Compliance letter is included in the administrative binder .

For both Merck and non-Merck studies, the SPONSOR will promptly be advised of any regulatory agency inspection of the institution or IRB/IEC conducted within the past 2 years or during the course of this study. Final results (i.e., final observations and responses) of such inspections should be promptly communicated to the SPONSOR. Any “SPONSOR-related” confidential information (SPONSOR name, study titles, compound identifiers, etc.) should be redacted via correction fluid or other appropriate methods from communications regarding inspection results for non-Merck studies.

The SPONSOR will promptly be advised of any IRB/IEC inspection of the investigator for a Merck-sponsored study during the course of this trial. The notification and outcome [written report(s) and response(s) when available] of such inspections should be promptly communicated to the SPONSOR by the investigator. The investigator will maintain copies of all IRB/IEC inspection reports and responses in the corresponding study file(s).

## 3.5 Data Analysis

This section outlines the statistical analysis strategy and procedures for the study. No separate Statistical Analysis Plan (SAP) will be issued for this LTFU study.

### 3.5.1 Responsibility for Analyses/In-House Blinding

The statistical analysis of the data obtained from this study will be the responsibility of the Late Development Statistics department of the SPONSOR. As stated in Section , treatment assignment blinding of study subjects and personnel is not applicable to this LTFU study.

### 3.5.2 Hypotheses/Estimation

Objectives and hypotheses of the study are stated in Section 2.1.

### 3.5.3 Analysis Endpoints

The HPV-related disease endpoints that will be addressed in this LTFU study are as defined in Sections 2.5.2 and 2.5.3 of this protocol.

### 3.5.4 Analysis Populations

The analysis populations that will be examined in this LTFU study are the PPE, HNRT, and FAS populations that are described in Section 2.7 of this protocol.

### 3.5.5 Statistical Methods

Because there is no placebo group in this LTFU study, vaccine efficacy (VE) estimation as described in Section 3.3.1 of the V501-019 SAP does not apply to this LTFU study. In lieu of VE estimates, cumulative incidence probabilities (or percents) based on non-parametric life-table methods will be computed for each of Cohorts 1 and 2 as described in Section of this protocol. The cohort-specific cumulative incidence probabilities will be put into context against the GARDASIL™ group- and placebo group-specific cumulative incidences observed during the base study. Details are provided in Section of this protocol.

As stated in Section 2.7, three analyses are planned corresponding to the time when all participating subjects have completed each of the scheduled visits at Years 6, 8, and 10 relative to the base study Day 1. Figure 3-1 shows key analysis-related time points and time intervals relative to the Years 6, 8, and 10 analysis time points in the LTFU study. Section of this protocol describes the observational comparisons relating to effectiveness that are feasible within the context of this LTFU study based on the key time points and time intervals shown in Figure 3-1.

Figure 3-1

Schematic Representation of Key Analysis-Related Time Points and Time Intervals
Relative to the Years 6, 8, and 10 Analysis Time Points in the LTFU Study

#### 3.5.5.1 Statistical Methods for Assessment of Effectiveness

Effectiveness will be assessed by computing cohort-specific incidences during the LTFU study as shown in Figure 3-1 and these LTFU incidences will be put into context against the incidences observed during the base study as summarized in Table 3-4. The cumulative incidence probabilities (or percents) based on non-parametric life-table methods, not the incidence rate (per 100 person-years), will be the primary metric that will be used for computing the cumulative incidences that will be used in observationally assessing effectiveness as summarized in Table 3-4.

Cohort 1

- At the Year 6 time point, cumulative incidence from Day 1 to Year 6 of HPV 6/11/16/18-related disease endpoints will be computed and reported. There are no corresponding 6-year cumulative incidences from the base study. Also at the Year 6 timepoint, cumulative incidences (of HPV 6/11/16/18-related disease endpoints) from Year 4 to Year 6, representing a 2-year incidence, will be computed and reported. This 2-year incidence will be evaluated in the context of the 2-year incidence from Day 1 to Year 2, and from Year 2 to Year 4, observed during the base study. Note that a 2-year period in the context of accumulating HPV 6/11/16/18-related disease endpoints is a very short time, the incidence is expected to be low, and the estimate of the cumulative incidence is not expected to have a high degree of precision.
- The continuing effectiveness of vaccination in Cohort 1 at the Year 8 time point will be assessed by evaluating the 4-year incidence in Cohort 1 from Years 4 to 8 (see Figure 3-1 and Table 3-4) in the context of Cohort 1's 4-year incidence from Day 1 to Year 4 during the base study. Similar 4-year incidences from Day 1 to Year 4 and from Years 4 to 8 are an indication of continuing vaccine effectiveness.
- The continuing effectiveness of vaccination in Cohort 1 at the Year 10 time point will be assessed by evaluating the 4-year incidence in Cohort 1 from Years 6 to 10 in the context of Cohort 1's 4-year incidence from Day 1 to Year 4 of the base study, and Cohort 1's 4-year incidence from Year 4 to Year 8. Similar 4-year incidences from Day 1 to Year 4, from Years 4 to 8, and from Years 6 to 10 are an indication of continuing vaccine effectiveness.

Cohort 2

- At the Year 6 time point, Cohort 2 subjects would have had approximately only a 1-year follow-up relative to the date of their vaccine dose 1 injection. A 1-year follow-up time post-dose 1 is too short for the purpose of accumulating and observing HPV 6/11/16/18-related disease endpoints. No cumulative incidence summaries will be computed for Cohort 2 at the Year 6 time point.
- The effectiveness of vaccination in Cohort 2 at the Year 8 time point will be assessed by evaluating the incidence from vaccine dose 1 to Year 8, which for Cohort 2 represents a 3-year incidence post-dose 1 (see Figure 3-1 and Table 3-4), in the context of the GARDASIL™ group's 3-year incidence from Day 1 to Year 3 during the base study. The 3-year duration (instead of 4 years) for trying to capture and observe a HPV-related disease endpoint is a limitation of the Year 8 effectiveness assessment in Cohort 2.
- The effectiveness of vaccination in Cohort 2 at the Year 10 time point will be assessed by evaluating the 4-year incidence in Cohort 2 from Years 6 to 10 in the context of the GARDASIL group's 4-year incidence from Day 1 to Year 4 during the base study. Similar 4-year incidences from Day 1 to Year 4 in the base study GARDASIL™ group and from Years 6 to 10 in Cohort 2 is an indication of vaccine effectiveness in Cohort 2.

Table 3-4

Summary of Effectiveness Measures in the LTFU Study and the Corresponding
Incidence Measures in the Base Study
(By Analysis Time and Cohort)

| Analysis Time  Cohort | Incidence During LTFU Study | vs. | Incidence During Base Study |
| --- | --- | --- | --- |
| Year 6 |  |  |  |
| Cohort 1 | 6-year incidence (Day 1 to Year 6)  2-year incidence (Years 4 to 6) | vs.  vs. | N/A  2-year incidence^†^ in Cohort 1^§^; 2-year incidence^†^ in the Cohort 2^║^;  2-year incidence^†^ in the Placebo group. |
| Year 8 |  |  |  |
| Cohort 1 | 8-year incidence (Day 1 to Year 8)  4-year incidence (Years 4 to 8) | vs.  vs. | N/A  4-year incidence^‡^ in Cohort 1^§^; 4-year incidence^‡^ in the Cohort 2^║^;  4-year incidence^‡^ in the Placebo group. |
| Cohort 2 | 3-year incidence (Years 5 to 8) | vs. | 3-year incidence^¶^ in Cohort 2^║^; 3-year incidence^¶^ in Cohort 1;  3-year incidence^¶^ in the GARDASIL™ group. |
| Year 10 |  |  |  |
| Cohort 1 | 10-year incidence (Day 1 to Year 10)  4-year incidence (Years 6 to 10) | vs.  vs. | N/A  4-year incidence^‡^ in Cohort 1^§^; 4-year incidence^#^ in Cohort 1; 4-year incidence^‡^ in the Cohort 2^║^;  4-year incidence^‡^ in the Placebo group. |
| Cohort 2 | 5-year incidence (Dose 1 to Year 10)  4-year incidence (Years 6 to 10) | vs.  vs. | N/A  4-year incidence^‡^ in Cohort 2^║^; 4-year incidence^‡^ in Cohort 1;  4-year incidence^‡^ in the GARDASIL™ group. |
| ^†^ Cumulative incidence from Day 1 to Year 2; and also cumulative incidence from Year 2 to Year 4.  ^‡^ Cumulative incidence from Day 1 to Year 4.  ^§^ Subjects from Colombia randomized to the GARDASIL™ group.  ^║^ Subjects from Colombia randomized to the placebo group.  ^¶^ Cumulative incidence from Day 1 to Year 3.  ^#^ Cumulative incidence from Years 4 to 8.  LTFU = Long term follow-up; N/A = Not available. There is no corresponding incidence during the base study. | | | |

#### 3.5.5.2 Statistical Methods for Assessment of HPV type Replacement

Possible HPV type replacement will be studied in the cohort of adult women in Colombia. While HPV type-specific, population-based data are not available in Colombia, type replacement can be assessed by describing the proportion of non-vaccine HPV types that produce high grade lesions as a percent of total biopsies obtained within the region where Protocol 019 was conducted. The incidence of CIN 2+ due to non-vaccine types in Cohorts 1 and 2 will be descriptively compared to the incidence of HPV type-specific CIN 2+ from similarly aged women in the cohort study performed by Dr. Nubia Muñoz and colleagues [4] in Bogota, Colombia. CIN 2+ HPV types that will be descriptively studied are the 10 non-vaccine types tested in the base study, HPV 31/33/35/39/45/51/52/56/58/59.

#### 3.5.5.3 Statistical Methods for Assessment of Immunogenicity

The procedure for estimating geometric mean titers (GMTs) as described in Section 3.3.2 of the V501-019 SAP will be used. GMTs and corresponding 95% confidence intervals of HPV (6, 11, 16, and 18) will be summarized for the time points indicated in Table 3-5.

Table 3-5

Summary of Time Points Post-Vaccine Dose 1 For Which HPV (6, 11, 16, and 18) Titers Will Be Available from the Base Study and LTFU Study

| Cohort | Time from Vaccine Dose 1 (in Months) | | | | | | | | |
| --- | --- | --- | --- | --- | --- | --- | --- | --- | --- |
|  | 7 | 12 | 24 | 36 | 48 | 60 | 72 | 96 | 120 |
| GARDASIL™ Group (Base study) ^†^ | + | + | + | + | + |  |  |  |  |
| Cohort 1 (LTFU study)^†^ | + | + | + | + | + |  | + | + | + |
| Cohort 2 (LTFU study) |  | + |  | + |  | + |  |  |  |
| + indicates that HPV (6, 11, 16, and 18) titers will be available corresponding to the indicated time point in the indicated cohort at the end of the LTFU study.  ^†^ Cohort 1 includes subjects enrolled and vaccinated with GARDASIL™ on Day 1 in the base study who continued into LTFU.  LTFU = Long term follow-up. | | | | | | | | | |

### 3.5.6 Multiplicity

No hypothesis will be tested in this LTFU study. Therefore, multiplicity adjustment does not apply to this study.

### 3.5.7 Power and Sample Size

As stated in Section 2.7, the sample size for the effectiveness and immunogenicity components of the LTFU study is fixed by the number of Protocol 019 base study participants from Colombia who are eligible and willing to participate in this LTFU study. The expected number of cases that can potentially be observed during the LTFU study based on ~1,600 subjects, together with the number of cases observed in some endpoints during the base study, makes it difficult to statistically differentiate the incidences observed during the LTFU study with the comparator incidences identified in Table 3-4. As an example, Table 3-6 shows the incidences observed during the base study for some selected vaccine HPV type-related disease endpoints in the per-protocol efficacy and full analysis set populations. For the endpoint of HPV 16/18-related CIN 2/3 or worse in the per-protocol efficacy population, the 4-year cumulative incidence (based on life-table estimate) during the base study are as follows:

- 0% in Cohort 1 (i.e., Colombia subjects randomized to the GARDASIL™ group) (the asymptotic standard error of cumulative incidence based on life-table method is 0 due to zero cases observed, therefore, 95% CI of cumulative incidence is not estimable);
- 0.07% (95% CI: 0.01 to 0.47) in the GARDASIL™ group;
- 0.41% (95% CI: 0.19 to 0.92) in the placebo group;
- 0.47% (95% CI: 0.15 to 1.46) among Colombia subjects randomized to the placebo group (i.e., Cohort 2 subjects).

Thus, for the base study 4-year cumulative incidence of HPV 16/18-related CIN 2/3 or worse in the per-protocol efficacy population, the incidence in the GARDASIL™ group is not statistically differentiable from the incidence in the placebo group (either all placebo recipients or only placebo recipients from Colombia). Consequently, it will be difficult to statistically differentiate the 4-year cumulative incidence of HPV 16/18-related CIN 2/3 or worse in the per-protocol efficacy population in Cohort 1 during the LTFU study (either from Years 4 to 8 or Years 6 to 10) from the base study 4-year cumulative incidence in the placebo group (either all placebo recipients or only placebo recipients from Colombia).

Table 3-6

Life-Table Estimate of Cumulative Incidence of HPV 6/11/16/18-Related Disease Endpoints During the Base Study
(Per-Protocol Efficacy and Full Analysis Set Populations)

| Endpoint | Time from Vaccine Dose 1 | GARDASIL™  (N=1,910) | | | Placebo (N=1,907) | | |
| --- | --- | --- | --- | --- | --- | --- | --- |
|  |  | Cases | Cumulative Incidence | | Cases | Cumulative Incidence | |
|  |  |  | % | (95% CI) |  | % | (95% CI) |
| **Per-Protocol Efficacy Population** |  |  |  |  |  |  |  |
| HPV 6/11/16/18-related CIN or EGL |  |  |  |  |  |  |  |
| All base study subjects | Year 2 | 1 | 0.06 | (0.01, 0.45) | 11 | 0.70 | (0.39, 1.27) |
|  | Year 3 | 1 | 0.06 | (0.01, 0.45) | 18 | 1.16 | (0.74, 1.84) |
|  | Year 4 | 1 | 0.06 | (0.01, 0.45) | 23 | 4.37 | (2.46, 10.5) |
| Colombia cohort | Year 2 | 0 | 0.00 | — | 5 | 0.72 | (0.30, 1.72) |
|  | Year 3 | 0 | 0.00 | — | 12 | 1.74 | (1.00, 3.06) |
|  | Year 4 | 0 | 0.00 | — | 13 | 1.93 | (1.16, 3.46) |
| HPV 6/11-related EGL |  |  |  |  |  |  |  |
| All base study subjects | Year 2 | 0 | 0.00 | — | 4 | 0.31 | (0.12, 0.83) |
|  | Year 3 | 0 | 0.00 | — | 7 | 0.55 | (0.26, 1.15) |
|  | Year 4 | 0 | 0.00 | — | 7 | 0.55 | (0.26, 1.15) |
| Colombia cohort | Year 2 | 0 | 0.00 | — | 1 | 0.18 | (0.03, 1.27) |
|  | Year 3 | 0 | 0.00 | — | 4 | 0.72 | (0.27, 1.91) |
|  | Year 4 | 0 | 0.00 | — | 4 | 0.72 | (0.27, 1.91) |
| HPV 16/18-related CIN 2/3 or worse |  |  |  |  |  |  |  |
| All base study subjects | Year 2 | 1 | 0.07 | (0.01, 0.47) | 3 | 0.20 | (0.07, 0.63) |
|  | Year 3 | 1 | 0.07 | (0.01, 0.47) | 6 | 0.41 | (0.19, 0.92) |
|  | Year 4 | 1 | 0.07 | (0.01, 0.47) | 6 | 0.41 | (0.19, 0.92) |
| Colombia cohort | Year 2 | 0 | 0.00 | — | 1 | 0.15 | (0.02, 1.08) |
|  | Year 3 | 0 | 0.00 | — | 3 | 0.47 | (0.15, 1.46) |
|  | Year 4 | 0 | 0.00 | — | 3 | 0.47 | (0.15, 1.46) |
| **Full Analysis Set** |  |  |  |  |  |  |  |
| HPV 6/11/16/18-related CIN or EGL |  |  |  |  |  |  |  |
| All base study subjects | Year 2 | 31 | 1.67 | (1.18, 2.36) | 41 | 2.22 | (1.64, 3.01) |
|  | Year 3 | 36 | 1.95 | (1.41, 2.70) | 51 | 2.79 | (2.13, 3.66) |
|  | Year 4 | 39 | 2.13 | (1.58, 2.94) | 60 | 3.33 | (2.64, 4.37) |
| Colombia cohort | Year 2 | 12 | 1.53 | (0.87, 2.67) | 22 | 2.79 | (1.84, 4.20) |
|  | Year 3 | 16 | 2.06 | (1.26, 3.33) | 29 | 3.70 | (2.58, 5.27) |
|  | Year 4 | 18 | 2.32 | (1.47, 3.65) | 38 | 4.94 | (3.73, 6.99) |
| HPV 6/11-related EGL |  |  |  |  |  |  |  |
| All base study subjects | Year 2 | 6 | 0.32 | (0.15, 0.72) | 8 | 0.44 | (0.22, 0.88) |
|  | Year 3 | 7 | 0.38 | (0.18, 0.80) | 11 | 0.61 | (0.34, 1.10) |
|  | Year 4 | 8 | 0.44 | (0.23, 0.95) | 12 | 0.67 | (0.38, 1.18) |
| Colombia cohort | Year 2 | 2 | 0.26 | (0.06, 1.03) | 3 | 0.38 | (0.12, 1.18) |
|  | Year 3 | 3 | 0.39 | (0.13, 1.20) | 6 | 0.77 | (0.35, 1.71) |
|  | Year 4 | 3 | 0.39 | (0.13, 1.20) | 7 | 0.90 | (0.43, 1.89) |
| HPV 16/18-related CIN 2/3 or worse |  |  |  |  |  |  |  |
| All base study subjects | Year 2 | 17 | 0.94 | (0.58, 1.50) | 19 | 1.03 | (0.66, 1.61) |
|  | Year 3 | 21 | 1.17 | (0.76, 1.79) | 23 | 1.27 | (0.84, 1.90) |
|  | Year 4 | 21 | 1.17 | (0.76, 1.79) | 27 | 1.53 | (1.08, 2.30) |
| Colombia cohort | Year 2 | 7 | 0.90 | (0.43, 1.88) | 8 | 1.02 | (0.51, 2.02) |
|  | Year 3 | 10 | 1.32 | (0.71, 2.43) | 9 | 1.16 | (0.60, 2.20) |
|  | Year 4 | 10 | 1.32 | (0.71, 2.43) | 13 | 1.76 | (1.08, 3.28) |
|  |  |  |  |  |  |  |  |
| CIN 2/3 or worse Due to Any HPV Type |  |  |  |  |  |  |  |
| All base study subjects | Year 2 | 48 | 2.63 | (1.98, 3.47) | 34 | 1.86 | (1.33, 2.58) |
|  | Year 3 | 55 | 3.04 | (2.34, 3.93) | 38 | 2.09 | (1.52, 2.86) |
|  | Year 4 | 59 | 3.28 | (2.59, 4.29) | 48 | 2.75 | (2.15, 3.79) |
| Colombia cohort | Year 2 | 20 | 2.58 | (1.67, 3.96) | 13 | 1.67 | (0.97, 2.86) |
|  | Year 3 | 23 | 2.99 | (1.99, 4.46) | 15 | 1.94 | (1.18, 3.21) |
|  | Year 4 | 24 | 3.13 | (2.15, 4.77) | 22 | 3.06 | (2.13, 4.98) |
| N = Number of subjects randomized to the respective vaccination group who received at least 1 injection.  CI = Confidence interval; CIN = Cervical intraepithelial neoplasia; EGL = External genital lesions; HPV = Human papillomavirus. | | | | | | | |

## 3.6 Data Management

Information regarding Data Management procedures for this protocol will be provided by the SPONSOR.

### 3.6.1 Study Documentation and Records Retention

Government agency regulations and directives require that all study documentation pertaining to the conduct of a clinical trial must be retained by the investigator. They shall be retained until at least 2 years after the last approval of a marketing application in an International Conference on Harmonisation (ICH) region, or at least 2 years have elapsed since the formal discontinuation of clinical development of the investigational product. These documents should be retained for a longer period, however, if required by the applicable regulatory requirements or by an agreement with the sponsor. The SPONSOR will notify the investigator in writing when retention is no longer necessary.

### 3.6.2 Laboratory Results

All assay results will be entered into the MRL clinical database.

## 3.7 Biological Specimens

Information regarding biological specimens for this protocol will be provided by the SPONSOR.

### 3.7.1 Shipment

Properly labeled specimens will be shipped according to instructions summarized in the SPONSOR’s central laboratory manual. Inventory forms, tubes for freezing/shipping specimens, and shipping containers will be provided by the SPONSOR’s central laboratory.

# 4. ADMINISTRATIVE AND REGULATORY DETAILS

## 4.1 Confidentiality

### 4.1.1 Confidentiality of Data

For Studies Conducted Under the U.S. IND

Particular attention is drawn to the regulations promulgated by the Food and Drug Administration under the Freedom of Information Act providing, in part, that information furnished to clinical investigators and Institutional Review Boards will be kept confidential by the Food and Drug Administration only if maintained in confidence by the clinical investigator and Institutional Review Board.

For All Studies

By signing this protocol, the investigator affirms to the SPONSOR that information furnished to the investigator by the SPONSOR will be maintained in confidence and such information will be divulged to the Institutional Review Board, Ethics Review Committee, or similar or expert committee; affiliated institution; and employees only under an appropriate understanding of confidentiality with such board or committee, affiliated institution and employees. Data generated by this study will be considered confidential by the investigator, except to the extent that it is included in a publication as provided in the Publications section of this protocol.

### 4.1.2 Confidentiality of Subject/Patient Records

For All Studies

By signing this protocol, the investigator agrees that the SPONSOR (or SPONSOR representative), Institutional Review Board/Independent Ethics Committee (IRB/IEC), or Regulatory Agency representatives may consult and/or copy study documents in order to verify worksheet/case report form data. By signing the consent form, the subject/patient agrees to this process. If study documents will be photocopied during the process of verifying worksheet/case report form information, the subject/patient will be identified by unique code only; full names/initials will be masked prior to transmission to the SPONSOR.

For Studies Conducted Under the U.S. IND

By signing this protocol, the investigator agrees to treat all patient data used and disclosed in connection with this study in accordance with all applicable privacy laws, rules and regulations, including all applicable provisions of the Health Insurance Portability and Accountability Act and its implementing regulations, as amended from time to time. (“HIPAA”).

### 4.1.3 Confidentiality of Investigator Information

For All Studies

By signing this protocol, the investigator recognizes that certain personal identifying information with respect to the investigator, and all subinvestigators and study site personnel, may be used and disclosed for study management purposes, as part of a regulatory submissions, and as required by law. This information may include:

- name, address, telephone number, and email address;
- hospital or clinic address and telephone number;
- curriculum vitae or other summary of qualifications and credentials; and
- other professional documentation.

Consistent with the purposes described above, this information may be transmitted to the SPONSOR, and subsidiaries, affiliates and agents of the SPONSOR, in your country and other countries, including countries that do not have laws protecting such information. Additionally, the investigator’s name and business contact information may be included when reporting certain serious adverse events to regulatory agencies or to other investigators. By signing this protocol, the investigator expressly consents to these uses and disclosures.

For Multicenter Studies

In order to facilitate contact between investigators, the SPONSOR may share an investigator’s name and contact information with other participating investigators upon request.

## 4.2 Compliance with Law, Audit, and Debarment

By signing this protocol, the investigator agrees to conduct the study in an efficient and diligent manner and in conformance with this protocol; generally accepted standards of Good Clinical Practice; and all applicable federal, state, and local laws, rules and regulations relating to the conduct of the clinical study.

The Code of Conduct, a collection of goals and considerations that govern the ethical and scientific conduct of clinical investigations sponsored by Merck Sharp & Dohme Corp., a subsidiary of Merck & Co., Inc., is attached.

The investigator also agrees to allow monitoring, audits, Institutional Review Board/Independent Ethics Committee review, and regulatory agency inspection of trial-related documents and procedures and provide for direct access to all study-related source data and documents.

The investigator agrees not to seek reimbursement from subjects/patients, their insurance providers, or from government programs for procedures included as part of the study reimbursed to the investigator by the SPONSOR.

The Investigator shall prepare and maintain complete and accurate study documentation in compliance with Good Clinical Practice standards and applicable federal, state, and local laws, rules and regulations; and, for each subject/patient participating in the study, provide all data, and upon completion or termination of the clinical study submit any other reports to the SPONSOR as required by this protocol or as otherwise required pursuant to any agreement with the SPONSOR.

Study documentation will be promptly and fully disclosed to the SPONSOR by the investigator upon request and also shall be made available at the investigator’s site upon request for inspection, copying, review, and audit at reasonable times by representatives of the SPONSOR or any regulatory agencies. The investigator agrees to promptly take any reasonable steps that are requested by the SPONSOR as a result of an audit to cure deficiencies in the study documentation and worksheets/case report forms.

International Conference of Harmonization Good Clinical Practice guidelines (Section 4.3.3) recommend that the investigator inform the subject’s primary physician about the subject’s participation in the trial if the subject has a primary physician and if the subject agrees to the primary physician being informed.

According to European legislation, a SPONSOR must designate a principal or coordinating investigator (CI) to review the report (summarizing the study results) and confirm that to the best of his/her knowledge the report accurately describes conduct and results of the study. The SPONSOR may consider one or more factors in the selection of the individual to serve as the CI (e.g., thorough understanding of clinical trial methods, appropriate enrollment of subject/patient cohort, timely achievement of study milestones, availability of the CI during the anticipated review process).

The investigator will promptly inform the SPONSOR of any regulatory agency inspection conducted for this study.

Persons debarred from conducting or working on clinical studies by any court or regulatory agency will not be allowed to conduct or work on this SPONSOR’s studies. The investigator will immediately disclose in writing to the SPONSOR if any person who is involved in conducting the study is debarred, or if any proceeding for debarment is pending or, to the best of the investigator’s knowledge, threatened.

In the event the SPONSOR prematurely terminates a particular trial site, the SPONSOR will promptly notify that site’s IRB/IEC.

## 4.3 Compliance with Financial Disclosure Requirements

By signing this protocol, the investigator agrees to provide to the SPONSOR accurate financial information to allow the SPONSOR to submit complete and accurate certification and disclosure statements as required by U.S. Food and Drug Administration regulations (21 CFR Part 54). The investigator further agrees to provide this information on a Financial Disclosure/Certification Form that is provided by Merck Sharp & Dohme Corp., a subsidiary of Merck & Co., Inc. This requirement also extends to subinvestigators. The investigator also consents to the transmission of this information to Merck Sharp & Dohme Corp., a subsidiary of Merck & Co., Inc., in the United States for these purposes. This may involve the transmission of information to countries that do not have laws protecting personal data.

## 4.4 Quality Control and Quality Assurance

By signing this protocol, the SPONSOR agrees to be responsible for implementing and maintaining quality control and quality assurance systems with written SOPs to ensure that trials are conducted and data are generated, documented, and reported in compliance with the protocol, accepted standards of Good Clinical Practice, and all applicable federal, state, and local laws, rules and regulations relating to the conduct of the clinical study.

## 4.5 Compliance with Information Program on Clinical Trials for Serious or Life Threatening Conditions

Under the terms of The Food and Drug Administration Modernization Act (FDAMA), the SPONSOR of the study is solely responsible for determining whether the study is subject to the requirements for submission to the Clinical Trials Data Bank*,* <http://clinicaltrials.gov/>. Merck, as SPONSOR of this study, will review this protocol and submit the information necessary to fulfill this requirement. Merck entries are not limited to FDAMA mandated trials. Merck’s voluntary listings, beyond those mandated by FDAMA, will be in the same format as for treatments for serious or life-threatening illnesses. Information posted will allow patients to identify potentially appropriate trials for their disease conditions and pursue participation by calling a central contact number for further information on appropriate study locations and site contact information.

By signing this protocol, the investigator acknowledges that the statutory obligation under FDAMA is that of the SPONSOR and agrees not to submit any information about this study to the Clinical Trials Data Bank.

## 4.6 Publications

This study is intended for publication, even if terminated prematurely. Publication may include any or all of the following: posting of a synopsis online, abstract and/or presentation at a scientific conference, or publication of a full manuscript. The SPONSOR will work with the authors to submit a manuscript describing study results within 12 months after the last data become available, which may take up to several months after the last patient visit in some cases such as vaccine trials. However, manuscript submission timelines may be extended on OTC studies. For studies intended for pediatric-related regulatory filings, the investigator agrees to delay publication of the study results until the SPONSOR notifies the investigator that all relevant regulatory requirements on the study drug have been fulfilled with regard to pediatric-related regulatory filings. Merck will post a synopsis of study results for approved products on [www.clinicalstudyresults.org](http://www.clinicalstudyresults.org/) and [www.clinicaltrials.gov](http://www.clinicaltrials.gov/) by 12 months after the last patient's last visit or within 7 days of product approval in any major markets (United States, Europe or Japan), whichever is later. These timelines may be extended for products that are not yet marketed, if additional time is needed for analysis, to protect intellectual property, or to comply with confidentiality agreements with other parties. Authors of the primary results manuscript will be provided the complete results from the Clinical Study Report, subject to the confidentiality agreement.

For multicenter studies, subsequent to the multicenter publication (or after public disclosure of the results online at [www.clinicalstudyresults.org](http://www.clinicalstudyresults.org/) if a multicenter manuscript is not planned), an investigator and his/her colleagues may publish their data independently. In most cases, publication of individual site data does not add value to complete multicenter results, due to statistical concerns. In rare cases, publication of single site data prior to the main paper may be of value. Limitations of single site observations in a multicenter trial should always be described in such a manuscript.

Authorship credit should be based on 1) substantial contributions to conception and design, or acquisition of data, or analysis and interpretation of data; 2) drafting the article or revising it critically for important intellectual content; and 3) final approval of the version to be published. Authors should meet conditions 1, 2, and 3. Significant contributions to study execution may also be taken into account to determine authorship, provided that contributions have also been made to all three of the preceding authorship criteria. Although publication planning may begin before conducting the study, final decisions on authorship and the order of authors’ names will be made based on participation and actual contributions to the study and writing, as discussed above. The first author is responsible to defend the integrity of the data, method(s) of data analysis, and the scientific content of the manuscript.

The SPONSOR must have the opportunity to review all proposed abstracts, manuscripts, or presentations regarding this study 60 days prior to submission for publication/presentation. Any information identified by the SPONSOR as confidential must be deleted prior to submission. SPONSOR review can be expedited to meet publication timelines.

# 5. LIST OF REFERENCES

1. Munoz, N.; Hernandez-Suarez, G.; Mendez, F.; Molano, M.; Posso, H.; Moreno, V.; Murillo, R.; Ronderos, M.; Meijer, C.; Munoz, A. Persistence of HPV infection and risk of high-grade cervical intraepithelial neoplasia in a cohort of Colombian women. Br J Cancer. 2009; 110(7): 1184-90.
2. Dillner, J.; Arbyn, M.; Dillner, L. Translational mini-review series on vaccines: monitoring of human papillomavirus vaccination. Clin Exp Immuno. 2007 148(2):199-207.
3. Kalantari M, Garcia-Carranca A, Morales-Vazquez CD, Zuna R, Montiel DP, Calleja-Macias IE, et al. Laser capture microdissection of cervical human papillomavirus infections: copy number of the virus in cancerous and normal tissue and heterogeneous DNA methylation. Virology 2009;390(2):261-7.
4. Munoz N, Hernandez-Suarez G, Mendez F, Molano M, Posso H, Moreno V, et al. Persistence of HPV infection and risk of high-grade cervical intraepithelial neoplasia in a cohort of Colombian women. Br J Cancer 2009;100(7):1184-90.

# 6. APPENDICES

There are no appendices.

# 7. ATTACHMENTS

Merck Sharp & Dohme Corp., a Subsidiary of Merck & Co., Inc., Code of Conduct for Clinical Trials

Mandatory Regimen for Triage to Coposcopy and Definitive Therapy—Investigator Aids

Procedure for Handling Biopsies Collect Out of Context of Study (BOCS) in the Merck HPV Program

Procedure for Handling PAP Tests Collected Out of Context of Study (POCS) in the Merck HPV Program
